# Development of a SCGP-CGAN framework for bidirectional H&E–Masson’s Trichrome Virtual Staining

**DOI:** 10.1371/journal.pone.0361713

**Published:** 2026-11-05

**Authors:** Zhe Du, Mengyang Xi, Tian Tang, Ke Yang, Shuchang Xu, Zhifeng Qu

**Affiliations:** 1 School of Medical Technology and Engineering, Henan University of Science and Technology, Luoyang, China; 2 Henan Engineering Research Center of Digital Pathology and Artificial Intelligence Diagnosis, Luoyang, China; 3 The First Affiliated Hospital of Henan University of Science and Technology, Luoyang, China; Islamia University of Bahawalpur: The Islamia University of Bahawalpur Pakistan, PAKISTAN

## Abstract

In virtual staining of histopathological sections, deep learning-based methods continue to face challenges arising from the irreversible nature of chemical staining, morphological variations between consecutive tissue sections, and the limited availability of multi-staining paired data from the same tissue region. While existing unsupervised approaches partially alleviate the reliance on paired datasets, they often struggle to maintain consistent color reproduction and preserve fine tissue structures. To address this issue, we develop a task-oriented framework for unsupervised virtual staining, referred to as SCGP-CGAN, where “S,” “C,” “G,” and “P” denote skip connections, CBAM-based attention, WGAN-GP-based adversarial optimization, and perceptual constraints, respectively, while “CGAN” refers to the underlying CycleGAN framework. Here, perceptual constraints refer to the complementary SSIM, LPIPS, style, and color consistency constraints used to promote structural, perceptual, stylistic, and color consistency in the generated images. This framework systematically integrates and evaluates existing components to enhance structural fidelity and perceptual quality in virtual staining. Experimental results on real histopathological datasets indicate that SCGP-CGAN exhibits favorable performance compared with CycleGAN and conventional staining normalization approaches under the evaluated dataset setting, showing improvements in perceptual quality and multiple quantitative evaluation metrics. All experiments are conducted on a publicly available patch-level dataset, and no independent WSI-level or multi-center validation is included. Within this experimental setting, the study demonstrates the effectiveness of the proposed framework for bidirectional H&E–Masson’s trichrome virtual staining and provides a methodological reference for further research in digital pathology image analysis.

## 1. Introduction

In modern digital pathology and histopathological image analysis, tissue staining serves as an essential technique for visualizing microstructural features and characterizing tissue morphology. Different staining methods use specific dyes to highlight structural components such as cell nuclei, stroma, and collagen fibers [1]. However, conventional histochemical staining typically involves multiple steps, including sectioning, fixation, and staining. This process is time-consuming and highly dependent on operator experience and the stability of experimental conditions, which may affect workflow efficiency and result reproducibility [2,3]. In addition, variations in dye concentration, section thickness, and imaging parameters can lead to inconsistencies in color and texture across different batches of samples [4,5]. This limits the generalization of multi-staining analysis and computational pathology methods.

To address these limitations, virtual staining has been proposed as a deep learning-based image generation approach. It enables the synthesis of tissue images with specific staining styles from unstained or single-stained inputs, while reconstructing key visual characteristics of traditional staining without requiring chemical processing [6,7]. Compared with conventional workflows, virtual staining is non-destructive, involves a simplified pipeline, and offers improved reproducibility [8,9]. As a result, it has attracted considerable attention as a promising technique for stain translation in computational pathology research [10].

In the field of digital pathology, unsupervised methods based on generative adversarial networks (GANs) have been widely applied, primarily in two types of tasks. One is stain normalization [11,12], which aims to reduce color and texture variations caused by differences in batches or experimental conditions, thereby improving visual consistency without generating realistic staining effects. The other is true virtual staining [8,13], which generates virtual tissue sections with specific staining styles from unstained or single-stained images, while partially restoring tissue structures and micromorphological features. Existing CycleGAN-based methods are commonly used for cross-stain transformations. Through adversarial loss and cycle consistency constraints, they can preserve overall structure and edge information without requiring paired training data [14–17]. However, in true virtual staining tasks, these methods still exhibit certain limitations, including mode collapse, blurred details, and insufficient representation of microstructures. These issues are particularly evident in fine structures such as cell nuclei and collagen fibers [18].

To address the aforementioned issues, this study focuses on bidirectional virtual staining between hematoxylin-eosin H&E and Masson’s trichrome histopathological images. Built upon the CycleGAN framework, SCGP-CGAN is formulated as a task-oriented framework for unsupervised virtual staining that integrates several established architectural components and loss functions. In SCGP-CGAN, “S,” “C,” “G,” and “P” denote skip connections, CBAM-based attention, WGAN-GP-based adversarial optimization, and perceptual constraints, respectively, while “CGAN” refers to the underlying CycleGAN framework. In the generator, U-Net-style skip connections [19] are introduced to enhance the preservation of spatial details and low-level texture information. In addition, a convolutional block attention module (CBAM) [20] is embedded within the residual blocks to adaptively strengthen feature representations related to tissue structures. For optimization, adversarial and cycle-consistency losses are combined with several established constraints, including color loss [11], structural similarity (SSIM) loss [21], style loss [22], and learned perceptual image patch similarity (LPIPS) loss [23]. These losses jointly constrain the generated results in terms of color, structure, and perceptual quality. In addition, the discriminator is trained using Wasserstein GAN with gradient penalty (WGAN-GP) [24] to provide a Wasserstein-based adversarial objective with gradient regularization for cross-stain image translation.

The framework is further evaluated through quantitative comparisons and ablation experiments to examine its overall performance and the contributions of its major architectural and optimization components. The main contributions of this study are summarized as follows:

(1) In the virtual staining task, the experimental results indicate that the integrated framework improves structural reconstruction quality. Compared with the original CycleGAN, the SSIM increases from 0.9239–0.9241 to 0.9829–0.9831, while the PSNR increases from 15.131–15.289 dB to 24.277–24.703 dB. These results suggest improved structural preservation and image reconstruction quality in the generated images.
(2) The incorporation of skip connections and attention mechanisms contributes to improved feature representation and structural detail preservation. For example, after introducing concat skip connections, the SSIM increases from 0.9239–0.9241 to 0.9359–0.9361. With the further incorporation of CBAM, the SSIM increases to 0.9759–0.9761, while the LPIPS decreases to 0.167, indicating improved detail preservation.
(3) WGAN-GP-based adversarial training is incorporated to provide a Wasserstein-based adversarial objective for cross-stain image translation. By introducing a gradient penalty into the discriminator objective, the framework regularizes the discriminator by constraining its gradient behavior during training.

## 2. Materials and methods

### 2.1. Related work

In digital pathology, variations in staining protocols, imaging devices, and experimental batches can introduce substantial differences in image appearance and affect the robustness of computational analysis [25]. In addition, chemical staining of pathological sections is inherently irreversible. A single tissue section can typically undergo only one physical staining procedure. This limitation makes it difficult to obtain multiple staining results from the same tissue region while preserving identical structural information. Virtual staining provides a potential solution by enabling the generation of different staining styles from existing images. This improves staining reproducibility and facilitates multi-stain analysis without additional physical tissue processing. To address this issue, two main approaches have been developed: stain normalization and stain transfer. Stain normalization aims to standardize image appearance [11,12]. Stain transfer focuses on learning mappings between different staining domains [16,26]. Among learning-based approaches, generative adversarial networks (GANs) have been widely used for unpaired image-to-image translation because they can model nonlinear relationships between staining domains.Traditional stain normalization methods, including Reinhard [11], Macenko [12], and Vahadane [27], mainly rely on color statistics, optical density modeling, or stain decomposition to reduce inter-stain variation. Although computationally efficient, these methods primarily focus on color normalization and may have limited capability in modeling complex relationships between staining appearance and tissue structure. GAN-based approaches, including CycleGAN [17], StainGAN [6], CUT [28], and MUNIT [29], enable cross-domain stain translation without strictly paired images and provide greater flexibility for nonlinear stain mapping. In this study, Macenko and Vahadane are included as conventional reference methods, while CycleGAN, StainGAN, and CUT serve as GAN-based image-to-image translation baselines for the H&E–MT cross-stain translation task. Together, these methods provide reference results from both conventional stain transformation and GAN-based image-to-image translation approaches.

Despite these advances, CNN-based GAN frameworks may have limitations in modeling long-range dependencies and global structures in high-resolution pathological images. Convolutional architectures primarily emphasize local spatial features, while attention mechanisms can complement convolutional feature extraction by adaptively emphasizing informative spatial and channel-wise features [30,31]. In virtual staining tasks, stain transfer involves not only local texture mapping but also maintaining consistency between structural organization and staining patterns across larger spatial regions.

To enhance structural representation in virtual staining, supervised approaches such as StainNet [32], DSTGAN [33], and pix2pix [16] have incorporated feature-level supervision, cross-domain consistency, or paired image reconstruction. These methods differ in their data requirements and supervision strategies, with pix2pix relying on strictly paired images, whereas StainNet and DSTGAN use different forms of feature-level or cross-domain supervision. However, the reliance on paired or stronger correspondence constraints limits their direct applicability to unpaired H&E–MT translation. In this study, we therefore focus on an unpaired bidirectional translation setting.

Recent studies have further explored adaptive, Transformer-based, and diffusion-based architectures for computational pathology image translation and virtual staining. Xu et al. proposed an adaptive stain normalization framework to improve robustness across different staining distributions [34]. Reisenbüchler et al. introduced an unsupervised latent stain adaptation framework to enhance cross-domain generalization through latent feature adaptation [35]. Transformer-based approaches have been investigated to capture long-range contextual relationships and improve structural representation in virtual staining and histopathological image translation [36]. More recently, diffusion-based frameworks have been explored for unpaired and multi-domain virtual staining, offering an alternative generative paradigm to conventional GANs [37,38]. These studies demonstrate the growing diversity of architectures for virtual staining. However, differences in staining targets, datasets, supervision settings, and image correspondence make direct quantitative comparison across studies challenging. The present study focuses specifically on bidirectional H&E–MT virtual staining using unpaired, non-pixel-aligned tissue images.

Motivated by these considerations, this study develops SCGP-CGAN, an improved CycleGAN-based framework that incorporates structural and contextual feature modeling together with multiple complementary loss constraints for bidirectional H&E–MT virtual staining.

### 2.2. Methodology

#### 2.2.1. Dataset description.

This study uses the Stain Transfer in Histopathology Dataset. It was constructed and publicly released by Zingman et al. for evaluating image-to-image translation and stain transfer in histopathological images [26]. No additional manual preprocessing or customized patch extraction was applied. We strictly follow the official dataset split provided by the authors. The training and test sets are directly adopted from the original dataset without any further re-partitioning or modification.

Specifically, the H&E and MT image patches were derived from corresponding anatomical regions of liver tissue obtained from multiple mouse specimens. The H&E and MT images were acquired from close tissue slices of the same liver region, and image patches were subsequently extracted from the corresponding tissue regions within the WSIs. Therefore, the dataset provides approximate tissue-level correspondence between H&E and MT image patches rather than strict pixel-wise paired images. The training set contains approximately 10,000 image patches of size 256 × 256 pixels, extracted from WSIs for each staining modality. The test set consists of approximately 1,300 image patches extracted from WSIs originating from different histological studies, resulting in a data distribution that differs from the training set.

This experimental setup enables evaluation under cross-study distribution shifts (training vs. test), providing an assessment of the model’s robustness under varying data distributions. Future work may further explore more rigorous slide-level partitioning strategies to better evaluate generalization under more diverse staining conditions.

#### 2.2.2. General Model Architecture.

This study proposes an improved virtual staining method based on CycleGAN, referred to as SCGP-CGAN.

As shown in Fig 1, the model consists of two generators (G: A → B, F: B → A) and their corresponding discriminators (D_A_, D_B_), forming a bidirectional adversarial framework. The generators perform cross-domain stain mapping, while the discriminators distinguish between real and generated images. The model is trained in an unsupervised manner and enables unpaired stain transfer through the combination of adversarial and cycle consistency losses.

**Fig 1:**
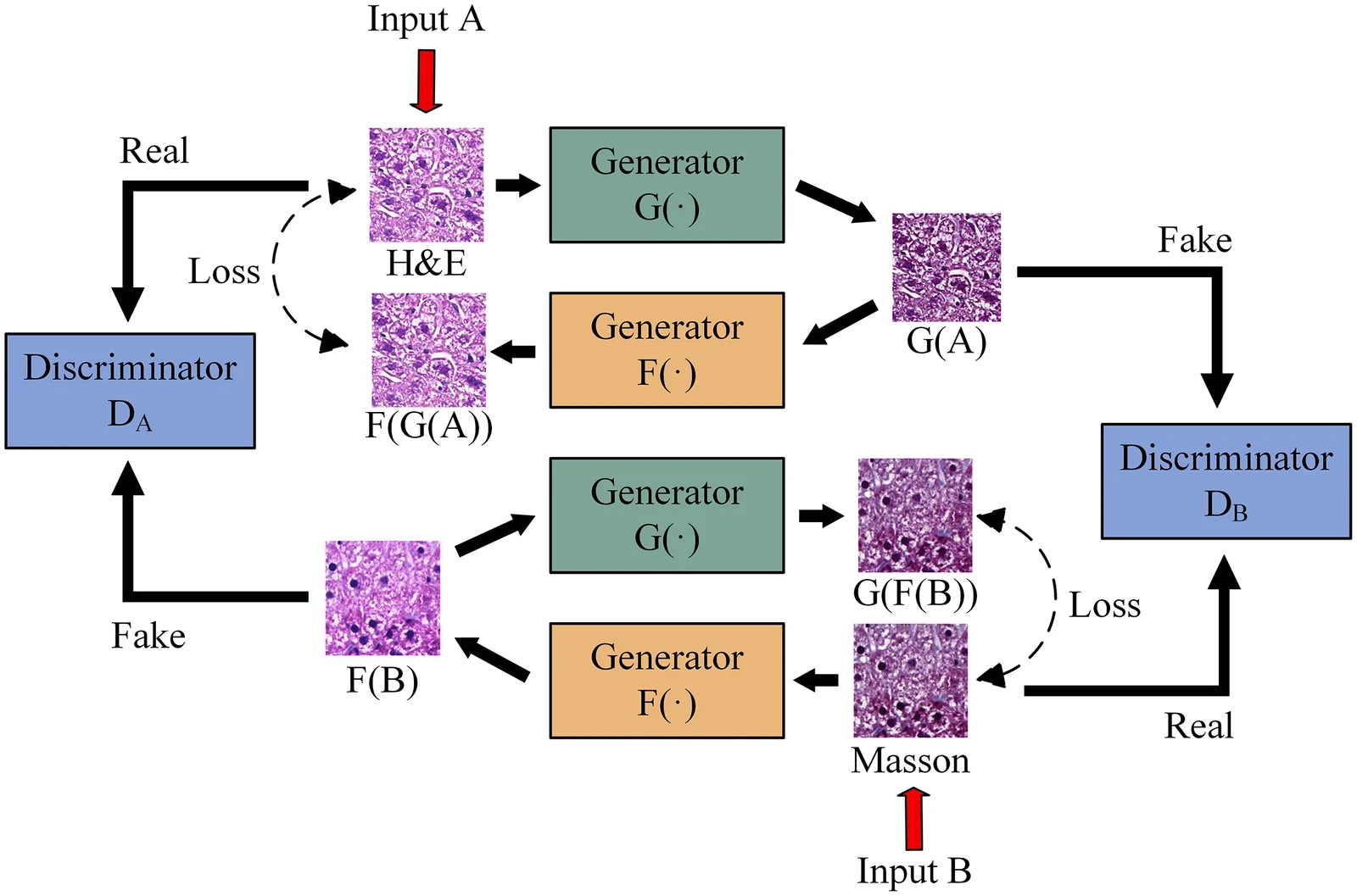
Overall architecture of the SCGP-CGAN model.

In terms of overall architecture, SCGP-CGAN incorporates several established architectural and optimization components to improve staining consistency and structural reconstruction. The generator adopts an encoder–decoder architecture and incorporates skip connections and attention mechanisms to enhance feature representation. The discriminator builds upon the PatchGAN framework and integrates Wasserstein adversarial optimization to improve training stability. In addition, the loss function combines multiple structural and perceptual constraints to guide the stain mapping process, with implementation details provided in subsequent sections.

#### 2.2.3. Generator Design.

In terms of the generator design, a ResNet-based generator architecture is adopted together with skip connections and the CBAM attention mechanism [39,40]. The integration of these components enables enhanced feature propagation and adaptive emphasis on informative regions. The overall architecture follows a symmetric encoder–residual module–decoder framework. As illustrated in Fig 2, this design enables hierarchical integration of semantic and spatial features within the feature flow.

**Fig 2:**
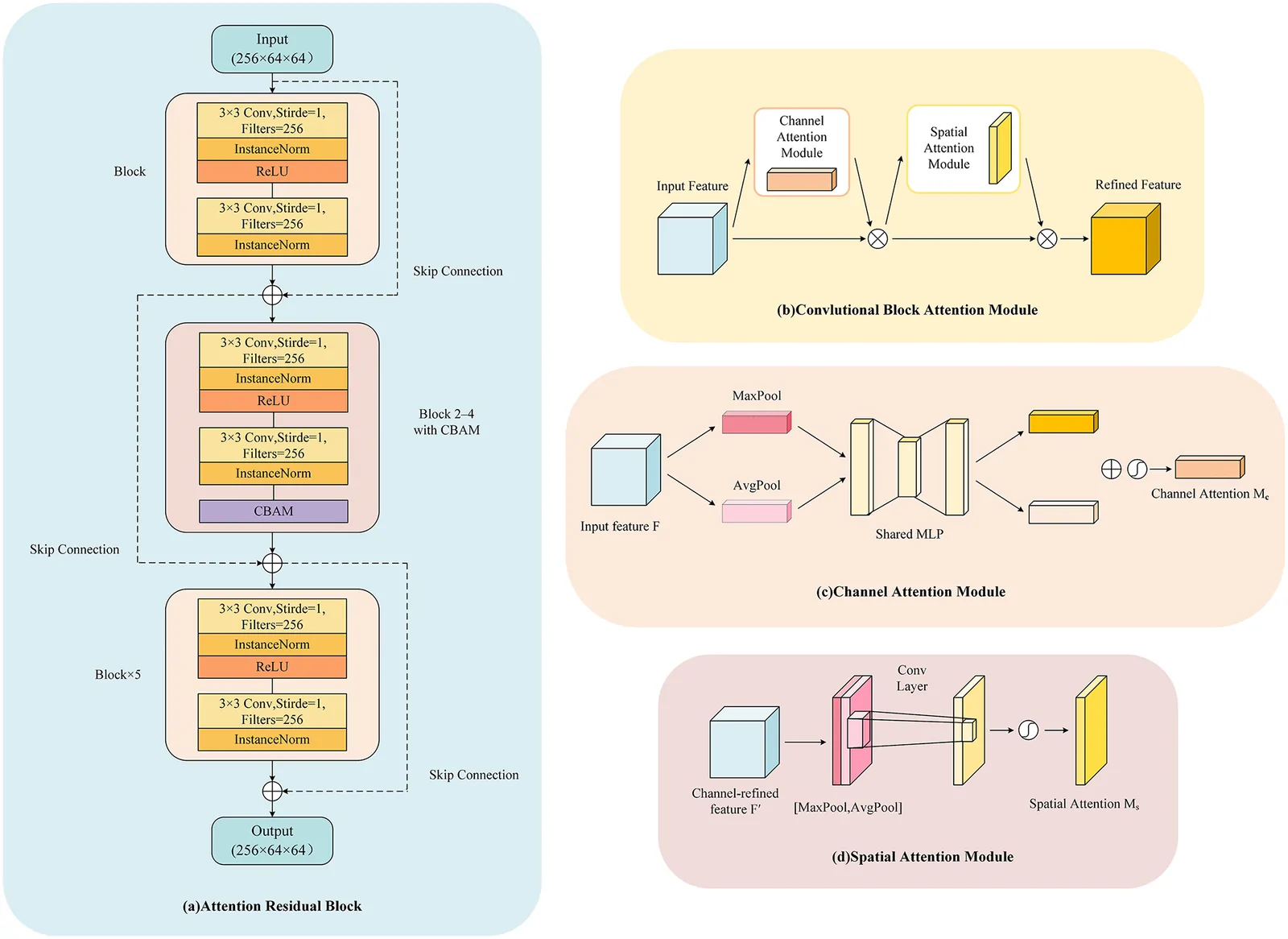
Schematic illustration of the improved generator architecture. (a) Attention residual block incorporating skip connections. (b) Overall structure of CBAM. (c) Channel attention component of CBAM. (d) Spatial attention component of CBAM.

CBAM is embedded within the residual modules. Each module consists of two 3 × 3 convolutional layers, instance normalization, and ReLU activation, with identity mapping used to facilitate feature fusion. The CBAM module reweights intermediate features along both channel and spatial dimensions, thereby modulating the feature response distribution. The core component comprises nine stacked residual blocks, which are responsible for modeling cross-domain mappings. Skip connections are introduced between corresponding layers of the encoder and decoder to mitigate information loss. In implementation, feature concatenation is adopted instead of element-wise addition. This allows shallow and deep features to be jointly modeled through subsequent convolutions, rather than directly bypassing deeper feature learning. As a result, over-reliance on shallow features is reduced. In addition, each residual block retains nonlinear transformations to ensure effective encoding of high-level semantic information.

As shown in Fig 3, the encoder extracts multi-scale features through successive convolution and downsampling operations: shallow layers primarily capture texture and edge information, while deeper layers encode higher-level semantic representations related to staining. The decoder progressively restores spatial resolution via upsampling and reconstructs features using a convolution–normalization–activation scheme.

**Fig 3:**
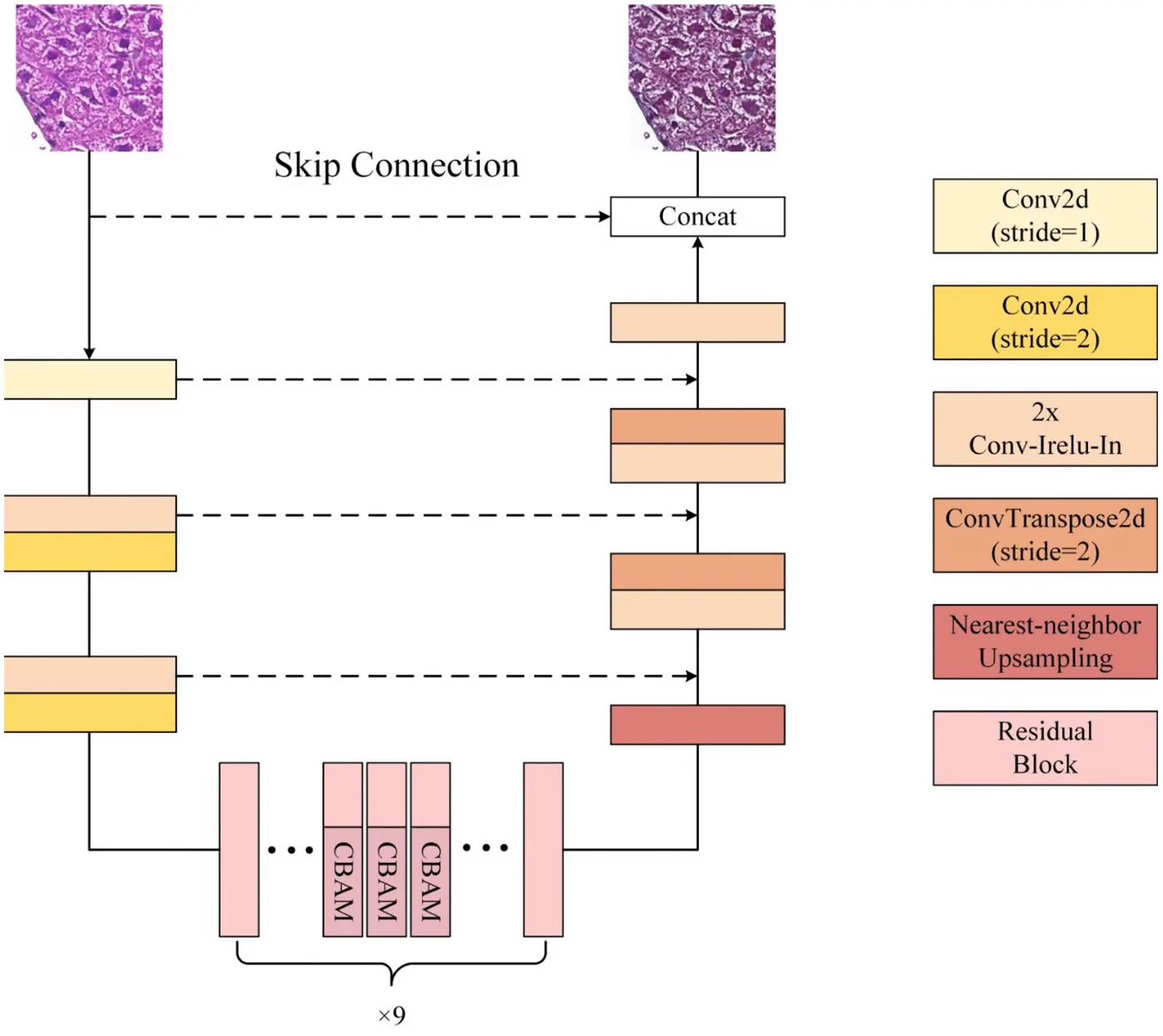
Schematic illustration of the improved residual module.

#### 2.2.4. Improved Discriminator Strategy.

This study constructs a discriminator based on the original PatchGAN architecture [16] and incorporates the gradient penalty mechanism from WGAN-GP [24]. Unlike the conventional PatchGAN, which outputs local realism probabilities, the discriminator is reformulated as a Critic network. The network produces real-valued scores for each input image and estimates the Wasserstein distance between the distributions of real and generated images. A gradient penalty is introduced into the discriminator objective to regularize the discriminator by constraining its gradient norm on interpolated samples. The WGAN-GP loss function is defined as follows:


$$\begin{array}{c} {\text{L}}_{\text{WGAN}-\text{GP}}=E_{\sim {\text{P}}_{\text{r}}}\left[\text{D}(\text{x})\right]-E_{\tilde{x}\sim {\text{P}}_{\text{g}}}\left[\text{D}\left(\tilde{x}\right)\right]+\lambda E_{\hat{\text{x}}\sim {\text{P}}_{\hat{\text{x}}}}\left[{\left({\left\|\nabla_{\hat{\text{x}}}\text{D}\left(\hat{\text{x}}\right)\right\|}_{\text{2}}-\text{1}\right)}^{\text{2}}\right] \end{array}$$ (1)

Here, P_r_ and P_g_ denote the distributions of real and generated images, respectively, where x represents a real sample and $\tilde{x}$ represents a generated sample. x̂ is a linearly interpolated sample between x and $\tilde{x}$, and λ represents the gradient penalty coefficient. The gradient penalty constrains the gradient norm of the Critic at the interpolated sample to be close to 1, enforcing the 1-Lipschitz condition and preventing gradient explosion or vanishing. Structurally, the discriminator retains the local receptive field design of PatchGAN to preserve its ability to discriminate local texture features, while the Sigmoid activation is removed and a linear score is output directly. In terms of training strategy, the generator and discriminator are updated with a 1:1 ratio. Although WGAN-GP commonly uses multiple Critic updates [24], a 1:1 update scheme was adopted in this study, consistent with the update strategy commonly used in CycleGAN and pix2pix [16,17]. The 1:1 strategy was adopted to maintain the same update frequency for the Critic and generator under the current experimental setting. As illustrated in Fig 4, the original PatchGAN produces local probability scores through a Sigmoid function and is optimized using LSGAN loss, primarily focusing on local realism. In contrast, after incorporating WGAN-GP, the Critic outputs real-valued scores without a Sigmoid function, and a gradient penalty is added to its objective. This formulation provides a Wasserstein-based adversarial objective together with gradient regularization, while retaining the local discriminative capability of the PatchGAN architecture.

**Fig 4:**
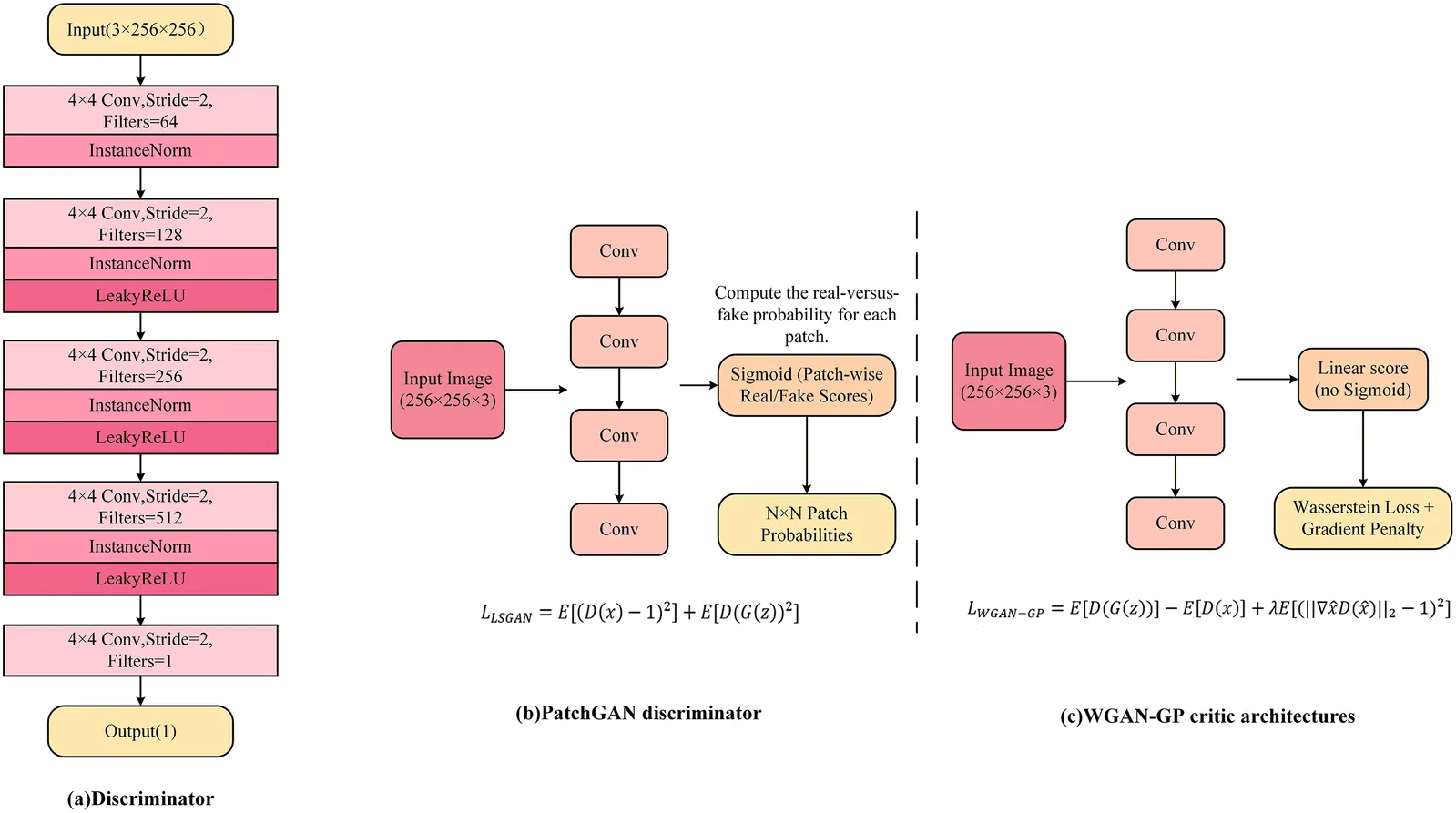
Schematic comparison of discriminator architectures.

#### 2.2.5. Loss function.

Building upon the optimized generator and discriminator architectures, this study incorporates multiple loss functions into the generator, including LPIPS perceptual loss, SSIM structural similarity loss, as well as style and color consistency constraints. These losses are introduced to improve the visual quality of the generated images and to better capture their structural and textural characteristics. The loss terms provide complementary constraints at different representational levels, targeting perceptual feature similarity, structural fidelity, and global texture consistency. The mathematical formulations and corresponding roles of these loss functions are described as follows.

LPIPS Loss [23]: By employing pre-trained deep networks to evaluate differences between generated and target images in the perceptual space, the similarity as perceived by human vision can be more accurately captured. Let ${\text{F}}_{\text{l}}(\text{x})\;$denote the feature map at layer lof the pre-trained network, then:


$$\begin{array}{c} {\text{L}}_{\text{LPIPS}}(\text{x},\text{y})=\sum_{\text{l}}\frac{\text{1}}{{\text{H}}_{\text{l}}{\text{W}}_{\text{l}}}\sum_{\text{h},\text{w}}||{\text{w}}_{\text{l}}⨀(\hat{{\text{F}}_{\text{l}}}(\text{x}{)}_{\text{h},\text{w}}-\hat{{\text{F}}_{\text{l}}}(\text{y}{)}_{\text{h},\text{w}})|{|}_{\text{2}}^{\text{2}} \end{array}$$ (2)

Here, x and ydenote the two input images. l denotes the index of the feature extraction layer. ${\hat{F}}_{l}$ denotes the normalized feature representation extracted from the l-th layer. $H_{l}$ and $W_{l}$ represent the spatial height and width of the l-th feature map. h,w are the spatial position indices. $w_{l}$ represents the corresponding learned channel weight vector. ${\hat{F}}_{l}(\cdot {)}_{h,w}$represents the channel vector at the specific spatialposition (h,w).

SSIM Loss [21]: Quantifies the similarity between generated and real images with respect to luminance, contrast, and structural information, thereby preserving the consistency of tissue architecture.

The SSIM is defined as:


$$\begin{array}{c} \text{SSIM}(\text{x},\text{y})=\frac{\left(\text{2}\mu_{\text{x}}\mu_{\text{y}}+{\text{C}}_{\text{1}})(\text{2}\sigma_{\text{xy}}+{\text{C}}_{\text{2}}\right)}{\left(\mu_{\text{x}}^{\text{2}}+\mu_{\text{y}}^{\text{2}}+{\text{C}}_{\text{1}})(\sigma_{\text{x}}^{\text{2}}+\sigma_{\text{y}}^{\text{2}}+{\text{C}}_{\text{2}}\right)} \end{array}$$ (3)

where x and y denote the generated and reference images, respectively; $\mu_{x}$ and $\mu_{y}$ are the local mean intensities; $\sigma_{x}^{\text{2}}$ and $\sigma_{y}^{\text{2}}$ are the local variances; and $\sigma_{xy}$ is the local covariance between the two images. $C_{\text{1}}$ and $C_{\text{2}}$ are stabilization constants used to improve numerical stability. SSIM values closer to 1 indicate greater structural similarity. Accordingly, the SSIM loss was defined as:


$$\begin{array}{c} {\text{L}}_{\text{SSIM}}(\text{x},\text{y})=\text{1}-\text{SSIM}(\text{x},\text{y}) \end{array}$$ (4)

Style Loss [22]: By exploiting Gram matrix–based representations to capture texture and style statistics, the style loss constrains the generator to produce images whose staining characteristics remain faithful to the target staining domain


$$\begin{array}{c} {\text{G}}_{\text{l}}(\text{x})={\text{F}}_{\text{l}}(\text{x}){\text{F}}_{\text{l}}(\text{x}{)}^{\text{T}} \end{array}$$ (5)

$$\begin{array}{c} {\text{L}}_{\text{style}}(\text{x},\text{y})=\sum_{\text{l}}\frac{\text{1}}{{\text{C}}_{\text{l}}^{\text{2}}}|\left|{\text{G}}_{\text{l}}(\text{x})-{\text{G}}_{\text{l}}(\text{y})\right|{|}_{\text{F}}^{\text{2}} \end{array}$$ (6)

where ${\text{F}}_{\text{l}}(\text{x})$ denotes the feature map of input image x extracted from the selected feature layer l, ${\text{G}}_{\text{l}}(\text{x})$ represents the corresponding Gram matrix used to characterize the feature correlations and capture the style information of the image. Here, l indicates the selected feature layer used for style representation.

Color Constancy Loss [11]: By constraining the balance of color distribution, overall color bias in the generated images can be avoided, ensuring consistency across different staining domains.


$$\begin{array}{c} {\text{L}}_{\text{color}}(\text{x})=\sum_{\left(\text{i},\text{j})\varepsilon \{(\text{R},\text{G}),(\text{R},\text{B}),(\text{G},\text{B})\right\}}(\frac{\mu_{\text{i}}(\text{x})}{\mu_{\text{j}}(\text{x})}-\text{1}{)}^{\text{2}} \end{array}$$ (7)

Here, x denotes the input image. $\mu_{i}(x)$ represents the spatial mean of the image x in the i-th color channel (where i∈{R,G,B}). The summation iterates over all distinct pairs of color channels (i,j)∈{(R,G),(R,B),(G,B)}.Overall Loss:


$$\begin{array}{c} {\text{L}}_{\text{total}}={\text{L}}_{\text{GAN}}+\lambda_{\text{cyc}}{\text{L}}_{\text{cyc}}+\lambda_{\text{id}}{\text{L}}_{\text{id}}+\lambda_{\text{LPIPS}}{\text{L}}_{\text{LPIPS}}+\lambda_{\text{SSIM}}{\text{L}}_{\text{SSIM}}+\lambda_{\text{style}}{\text{L}}_{\text{style}}+\lambda_{\text{color}}{\text{L}}_{\text{color}} \end{array}$$ (8)

During the staining translation process, a multi-loss combination is computed between the generated images and the corresponding target-domain images (fake H&E versus real H&E, and fake MT versus real MT) to enhance structural consistency and perceptual similarity. Although adjacent tissue sections are not strictly pixel-aligned, they still preserve certain similarities in tissue morphology, cellular distribution, staining patterns, and tissue structural characteristics. The constraints associated with SSIM and LPIPS mainly provide perceptual and statistical guidance for the staining translation process, enabling the generated results to be closer to the target staining domain in terms of perceptual features, texture representation, and staining style. Style Loss constrains the generated images to better match the statistical style distribution of the target staining domain in the feature space, relying more on high-level texture and appearance representations rather than precise spatial alignment. Color Loss is used to constrain the global stain color distribution of the generated images toward the target staining domain. Therefore, the introduction of the multi-loss combination jointly constrains the generated results from multiple aspects, including structure, perception, texture style, and color distribution, thereby improving the overall quality of the generated images in terms of tissue structure preservation, texture representation, and staining-style consistency.

Overall, these loss terms are not redundant; instead, they provide complementary constraints across local structure, perceptual features, and global texture. To balance the trade-offs among them, weight coefficients are introduced and validated through ablation studies and sensitivity analysis.

In this framework, the weight coefficient λ for each loss function is treated as an adjustable hyperparameter that controls the relative contribution of each constraint during optimization. Based on comparative experiments and parameter tuning, the selected configuration is $\lambda_{\text{LPIPS}}$=0.2, $\lambda_{\text{SSIM}}$=0.15, $\lambda_{\text{color}}$=3.0, $\lambda_{\text{style}}$=1.5. This setting provides a balance among perceptual consistency, structural fidelity, and color representation, while preserving fine histological details. The contribution and combined effect of each loss term are further analyzed in the subsequent ablation study (see Table 6).

### 2.3. Training strategy and hyperparameter configuration

All experiments were conducted in a Python 3.11 environment using PyTorch 2.1.0 and CUDA 11.8 on an NVIDIA GeForce RTX 4090 GPU with 24 GB of VRAM. The major packages used in this work include NumPy (v2.0.1), OpenCV (v4.11.0), Matplotlib (v3.10.1), and torchvision (v0.20.1), along with standard Python libraries such as os and shutil. The Adam optimizer (β_1_ = 0.5, β_2_ = 0.999) was employed with an initial learning rate of 2 × 10 ^−^ ⁴, which was kept constant throughout training. To improve reproducibility, a fixed global random seed (42) was used for all stochastic operations. All experiments were independently repeated five times to reduce variability and obtain stable quantitative results. The final model checkpoint was selected from the last training epoch, without additional validation-based selection.

In comparative experiments using batch sizes of 2 and 8, only minor differences were observed in key evaluation metrics. Considering that smaller batch sizes reduce VRAM usage and improve training efficiency, a batch size of 2 was selected. The training protocol follows the original CycleGAN, without introducing additional learning rate scheduling or regularization strategies. The number of training epochs was determined based on prior work [26] and empirical convergence behavior, with the model reaching stable convergence at approximately 40 epochs. During training, the loss curves exhibited stable convergence, with no noticeable oscillations or mode collapse. Repeated experiments yielded consistent results, indicating good training stability and reproducibility. Under the specified hardware conditions, a complete training run requires approximately 4 hours (about 6 minutes per epoch), and the computational cost remains within a typical range for comparable image translation tasks.

The loss weights λ were determined using an empirical, controlled adjustment strategy. During parameter tuning, other weights were held constant, and univariate analyses were performed on key parameters (e.g., SSIM and LPIPS) within reasonable ranges. The default weight configuration was selected based on the overall performance across multiple metrics, including structural similarity, perceptual quality, and distribution consistency. The corresponding sensitivity analysis results are presented in Tables 8 and 9.

To further assess the validity of the model configuration, ablation studies were conducted, including comparisons of different loss combinations and the removal of skip connections or attention modules. The results indicate that each component contributes consistently to overall performance and exhibits similar trends across different configurations. In combination with the sensitivity analysis, these findings suggest that the model maintains relatively stable performance within a reasonable parameter range, and that its effectiveness is not dependent on a specific parameter setting.

## 3. Results

### 3.1. Evaluation Metrics

To quantitatively evaluate the quality of virtual staining images, this study employs a set of complementary metrics, including PSNR [21], SSIM, MS-SSIM [41], LPIPS, FSIM [42], and FID [43].

As shown in Fig 5, the figure illustrates the image sources and the corresponding relationships between different evaluation metrics in the bidirectional virtual staining task. In the H&E → MT direction, real H&E images are used as inputs to generate fake MT images; conversely, in the MT → H&E direction, real MT images are used as inputs to generate fake H&E images.

**Fig 5:**
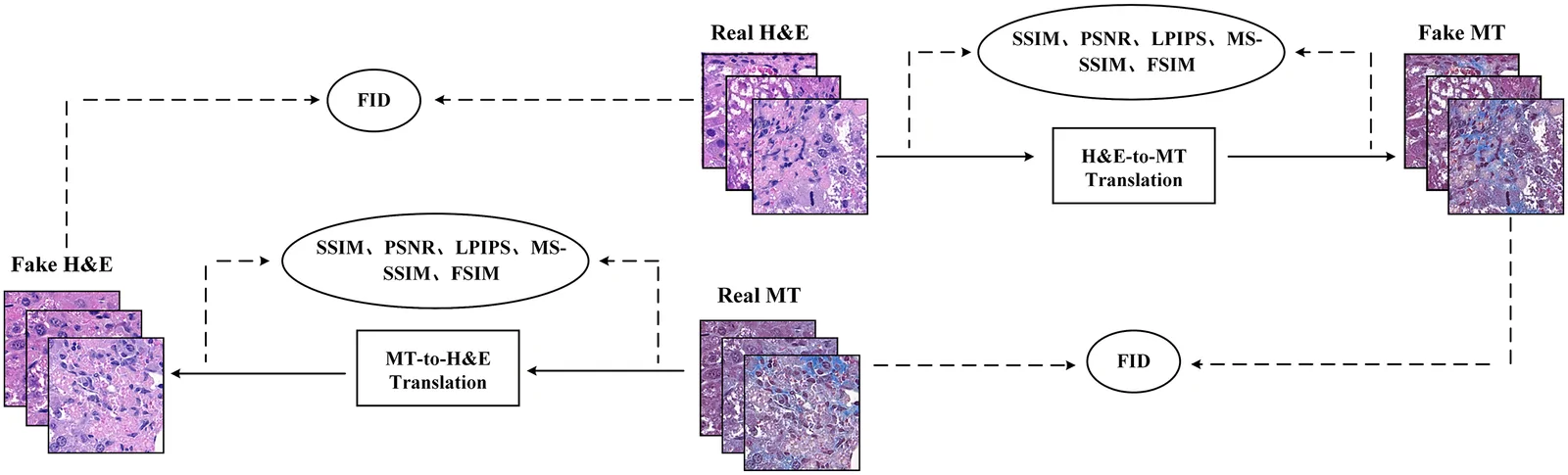
Schematic illustration of the image sources and comparison relationships used for different evaluation metrics in virtual staining assessment.

It should be noted that the real H&E and real MT images were obtained from adjacent tissue slices rather than the exact same physical tissue section. Therefore, no strict pixel-wise spatial correspondence exists between different staining modalities. However, during the virtual staining process, Fake MT is directly translated from the corresponding Real H&E image, while Fake H&E is directly translated from the corresponding Real MT image. As a result, the generated images are expected to preserve the same tissue structures and spatial layouts as their corresponding input images.

Based on this characteristic, SSIM, PSNR, LPIPS, MS-SSIM, and FSIM are used to evaluate the structural consistency and perceptual similarity between generated images and their corresponding source images. The comparisons include Real H&E versus Fake MT, as well as Real MT versus Fake H&E. These metrics are mainly used to assess the preservation of tissue structural information and perceptual features during the virtual staining process. They also reflect the degree of structural information degradation after stain translation, rather than pixel-level reconstruction accuracy across different staining domains.

Specifically, PSNR and SSIM are used to evaluate reconstruction fidelity and structural similarity between the generated images and the corresponding input images. MS-SSIM is employed to measure structural consistency across multiple scales. LPIPS and FSIM evaluate perceptual similarity based on deep perceptual features and phase congruency, respectively. In addition, FID is used to assess the feature-space distribution consistency between the generated image set and the real target-stained image set. This metric primarily reflects overall staining style consistency and cross-domain color distribution consistency.

For each metric, the evaluation is performed separately for the H&E → MT and MT → H&E translation directions using the complete set of generated test images from the testing dataset. The quantitative results reported in the manuscript correspond to the averaged performance of the two translation directions. To further assess experimental stability, all experiments were independently repeated five times. PSNR and SSIM are computed over five independent runs. The final reported values are presented as 95% confidence intervals derived from the mean and standard deviation of these runs, and the same representation is consistently used across all quantitative and ablation tables.To ensure correct interpretation of all evaluation metrics, directional indicators (↑/↓) are consistently provided in all tables and captions, where ↑ denotes that higher values indicate better performance and ↓ denotes that lower values indicate better performance.

### 3.2. Comparison with Different Methods

#### 3.2.1. Quantitative Comparison with Different Methods.

For comparison, three learning-based image-to-image translation methods, including CycleGAN, StainGAN, and CUT, were selected as learning-based baselines for the H&E–MT cross-stain translation task. Macenko and Vahadane were included as conventional stain normalization methods to provide reference results from traditional stain transformation approaches. All comparison methods were re-implemented using publicly available open-source repositories. All comparison methods used the same dataset and the same training and testing split, while the GAN-based models were trained with a batch size of 2. Different models adopted different numbers of training epochs according to the settings reported in the corresponding reference studies and preliminary experimental validation. Specifically, SCGP-CGAN, CycleGAN, and StainGAN were trained for 40 epochs, whereas CUT was trained for 30 epochs. In contrast, the traditional stain normalization methods, Macenko and Vahadane, do not require iterative adversarial training and were therefore applied in a single-pass manner. Other hyperparameter settings followed the default configurations provided in the original implementations.

No additional preprocessing, stain normalization, or image intensity normalization was applied during training or evaluation. Quantitative results for all GAN-based methods were obtained from five independent runs. For Macenko and Vahadane methods, no manually selected template image was used during evaluation.

As shown in Table 1, the quantitative results indicate that the proposed SCGP-CGAN generally outperforms the comparison methods across all evaluation metrics, demonstrating better structural fidelity and image quality consistency.

**Table 1:** Quantitative comparison of different models on the dataset.

Method category	Model	SSIM↑	PSNR↑	LPIPS↓	FID↓	MS-SSIM↑	FSIM↑
Conventional stain normalization	Macenko	(0.8960,0.8961)	(14.543,14.917)	0.191	96.14	0.819	0.818
	Vahadane	(0.8889,0.8891)	(13.964,14.276)	0.199	98.25	0.804	0.810
GAN-based image-to-image translation	CycleGAN	(0.9239,0.9241)	(15.131,15.289)	0.175	85.12	0.827	0.834
	StainGAN	(0.9199,0.9201)	(14.781,15.119)	0.184	88.13	0.825	0.818
	CUT	(0.9219,0.9221)	(14.898,15.242)	0.179	86.79	0.825	0.829
	SCGP-CGAN	**(0.9829,0.9831)**	**(24.277,24.703)**	**0.138**	**27.41**	**0.956**	**0.941**

In terms of structural similarity, SCGP-CGAN achieves SSIM scores of (0.9829, 0.9831). These values are higher than those of CycleGAN (approximately 0.924), StainGAN (approximately 0.920), as well as the traditional methods Macenko and Vahadane. This indicates that SCGP-CGAN has advantages in preserving structural information. Meanwhile, it obtains MS-SSIM and FSIM values of 0.956 and 0.941, respectively, further suggesting good performance in multi-scale structural consistency and perceptual quality. Regarding pixel-level reconstruction quality, SCGP-CGAN achieves PSNR values of 24.277–24.703 dB, higher than all comparison methods (most of which are around 14–15 dB), indicating that its generated results are closer to real images in terms of pixel distribution. For perceptual quality and distribution distance, SCGP-CGAN achieves an LPIPS value of 0.138, lower than CycleGAN (0.175), StainGAN (0.184), and traditional methods (up to 0.199), suggesting higher consistency with real images in deep feature space. In addition, its FID score is 27.41, lower than CycleGAN (85.12), StainGAN (88.13), and traditional methods (>80), indicating a smaller gap between the generated and real data distributions. Overall, GAN-based methods (CycleGAN, StainGAN, CUT) generally outperform traditional stain normalization methods (Macenko, Vahadane) across most metrics, but still fall behind the proposed method. Macenko and Vahadane show relatively weaker performance across all metrics, particularly in FID and LPIPS, indicating certain limitations in complex structure preservation and style transfer tasks.

In summary, SCGP-CGAN demonstrates stable performance in structural consistency, perceptual quality, and distribution matching, validating its effectiveness in virtual staining tasks.

#### 3.2.2. Qualitative comparison of generated images.

To intuitively evaluate the performance of different virtual staining methods, a qualitative comparative analysis is conducted in this section. The results of H&E-to-MT translation (Figs 6 and 7) and MT-to-H&E translation (Figs 8 and 9) are investigated separately.

**Fig 6:**
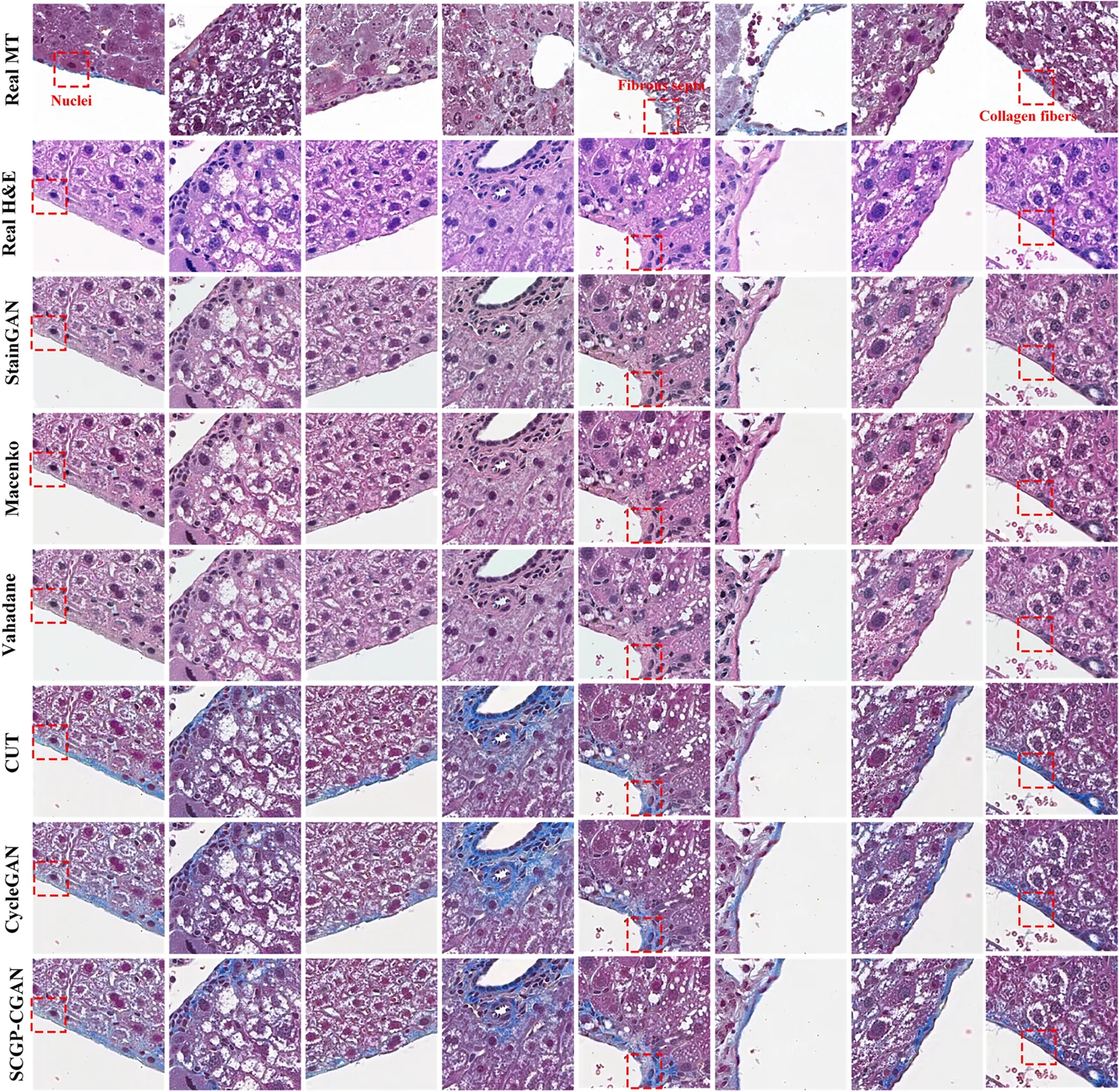
Qualitative comparison of different methods for H&E-to-Masson virtual staining, with annotated ROIs.

**Fig 7:**
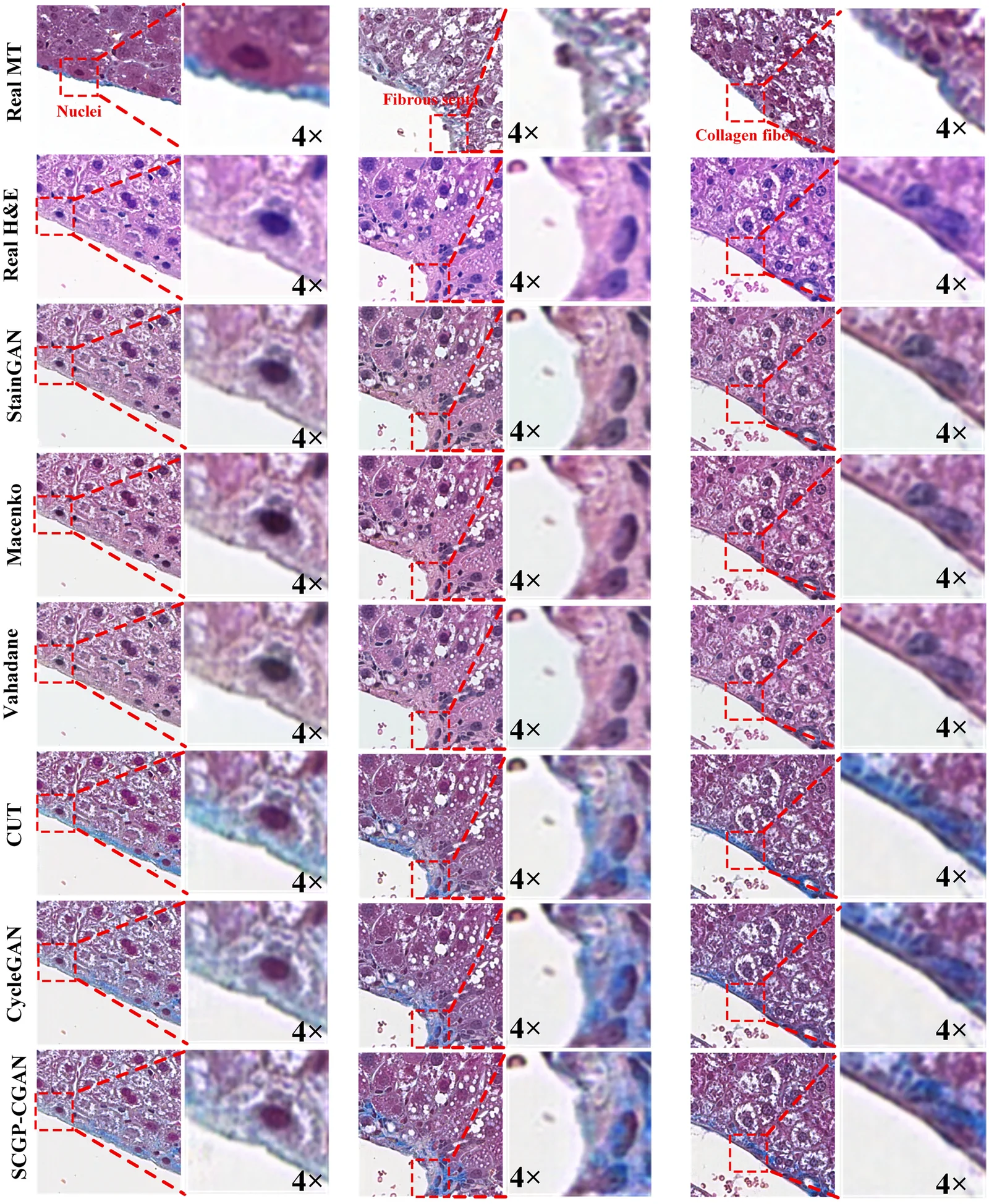
Magnified ROIs from Figure 6 for comparing histological structures across methods.

**Fig 8:**
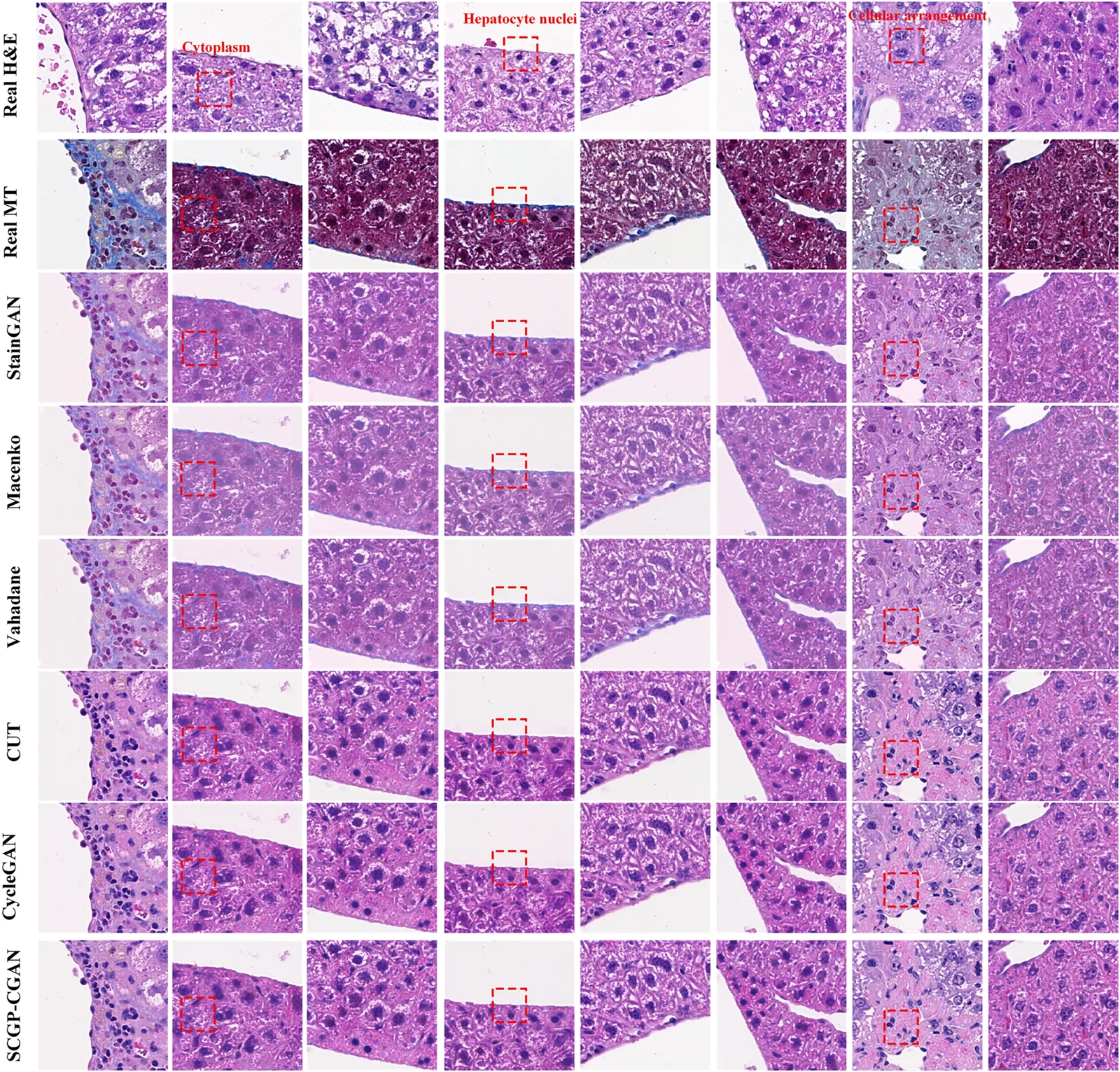
Qualitative comparison of different methods for Masson-to-H&E virtual staining, with annotated ROIs.

**Fig 9:**
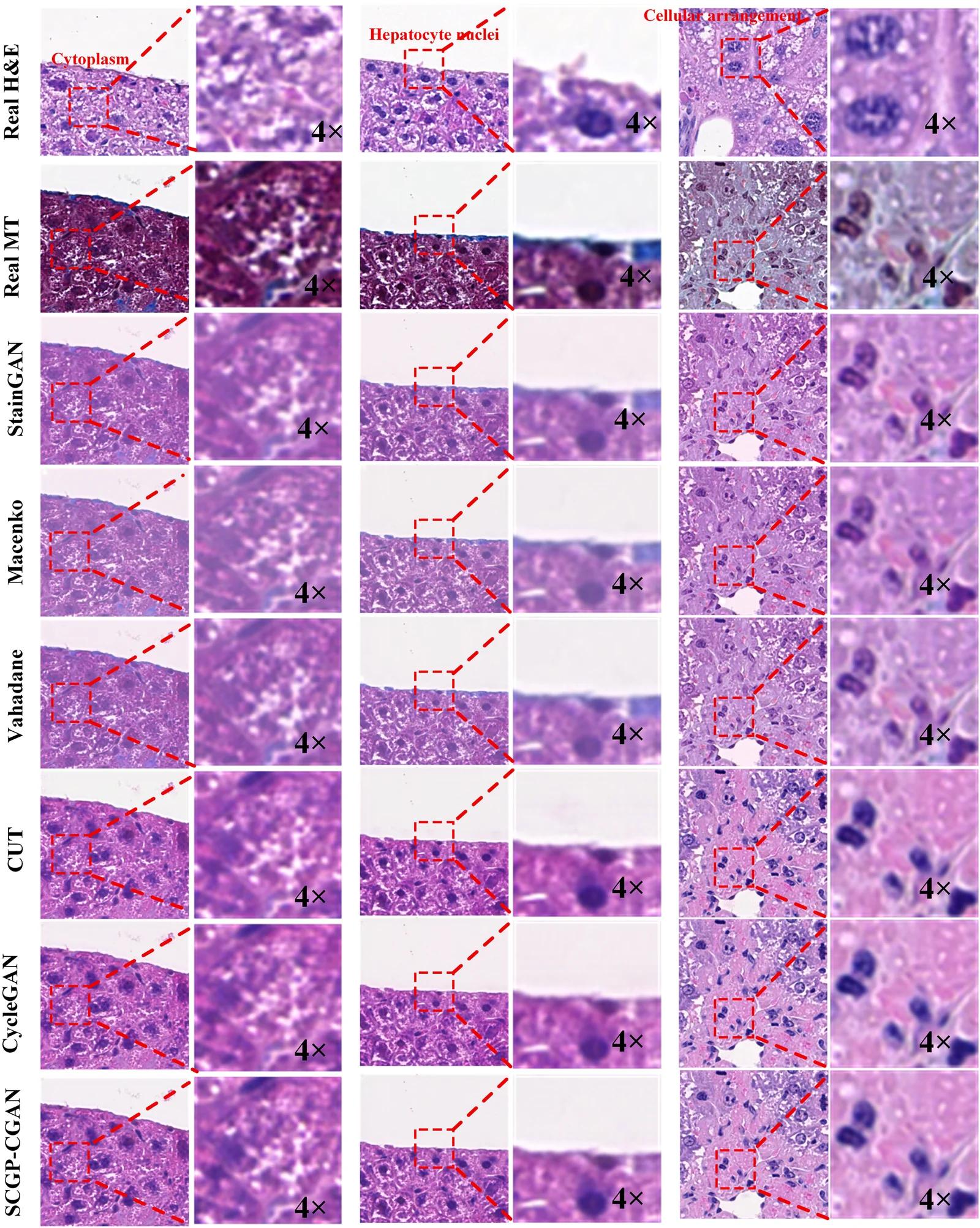
Locally magnified ROIs from Figure 8 for comparing histological structures across methods.

As shown in Fig 6, representative regions of interest (ROIs) are selected and magnified locally to highlight key histological structures, including cell nuclei, collagen fibers, cytoplasm, and cellular arrangement. These regions are selected with input from three experienced pathologists to improve the reliability and interpretability of the analysis.

Fig 6 presents the overall visual comparison among real MT, input H&E images (Real H&E), and the corresponding results generated by StainGAN, Macenko, Vahadane, CUT, CycleGAN, and SCGP-CGAN. Key histological structures are annotated to facilitate visual assessment. Fig 7 provides the corresponding locally magnified views of the annotated regions in Fig 6, allowing a more detailed inspection of fine-grained structures. The selected regions emphasize important histological components, including cell nuclei, fibrous septa, and collagen fibers, enabling a more comprehensive evaluation of structural preservation and staining consistency across different methods.

In terms of nuclear representation, StainGAN, Macenko, and Vahadane exhibit certain deviations in reproducing the characteristic staining of MT. In some results, the color saturation of nuclei is insufficient, the contrast with the surrounding cytoplasm is relatively weak, and the boundary clarity is reduced. In comparison, CycleGAN and CUT alleviate this issue to a certain extent but still suffer from local uneven coloration or unstable detail expression.

In fibrous septa and interstitial collagen regions, traditional methods (Macenko and Vahadane) show limited ability to recover the characteristic blue staining of MT. Some regions still retain H&E-like staining patterns, which results in low distinguishability of collagen structures. StainGAN can exhibit a blue staining trend in some regions, but its overall consistency remains unstable. CycleGAN and CUT can generate obvious blue regions, but problems such as color spreading, blurred boundaries, or uneven distribution may occur in local areas, thereby affecting the clear expression of collagen structures.

It should be noted that the staining intensity of collagen regions in real MT images inherently varies, which may be related to factors such as section thickness, tissue location, and staining batch. Collagen staining is weak in some real images. In such cases, SCGP-CGAN can combine structural information from the input H&E images to generate relatively consistent blue distribution, thus visually enhancing the identifiability of the relevant regions.

In terms of overall structural performance, StainGAN, Macenko, and Vahadane show relatively limited detail expression. Although CycleGAN and CUT achieve improved staining representation, slight structural discontinuities or uneven staining may appear in local regions. In contrast, SCGP-CGAN presents relatively stable structural representation in multiple key regions. In nuclear regions, the boundaries are relatively clear and the contrast with surrounding tissues is more natural; in fibrous septa regions, blue staining is mainly concentrated within the corresponding structures with coherent spatial distribution. Overall, the proposed method achieves a balanced performance between structural preservation and staining representation.

Fig 8 presents qualitative comparisons across different methods for the MT-to-H&E virtual staining task. The methods include StainGAN, Macenko, Vahadane, CUT, CycleGAN, and the proposed SCGP-CGAN. The results are shown alongside the corresponding real H&E and MT images. Fig 8 shows the overall visual comparison with annotated regions highlighting representative histological structures, while Fig 9 provides the corresponding locally magnified views of these annotated regions for more detailed observation. The selected regions focus on key histological features such as cytoplasm, hepatocyte nuclei, and cellular arrangement, enabling a more detailed evaluation of staining characteristics and structural fidelity across different methods.

From the overall visual effect, traditional stain normalization methods (Macenko and Vahadane) and StainGAN mainly achieve basic tone mapping. However, their performance in expressing the structural and textural features of H&E staining is relatively limited. Blurring or instability can be observed in nuclear boundaries, cytoplasmic details, and tissue arrangement. In contrast, GAN-based methods (CUT, CycleGAN) show improved overall tone restoration, but may still suffer from uneven staining, fluctuating detail expression, or insufficient structural continuity in local regions.

In terms of cytoplasmic performance, the cytoplasm in real H&E images shows a relatively uniform light pink or light purple tone with certain textural details. The results generated by some methods exhibit overly light or locally deep tones, accompanied by weakened textural details, which affects the contrast between the cytoplasm and nuclei. In comparison, SCGP-CGAN presents a relatively stable tone distribution in the cytoplasmic region and retains fine-grained textural information to a certain extent, making the overall visual effect closer to that of real staining.

For the restoration of hepatocyte nuclei, the nuclei in real H&E images typically appear dark purple with relatively clear boundaries. The nuclei generated by some methods show blurred boundaries, uneven staining, or local morphological deviations. CUT and CycleGAN achieve a certain consistency in nuclear position but may still exhibit slight halation artifacts. In contrast, SCGP-CGAN demonstrates a relatively stable morphological structure in the nuclear region, with boundary sharpness and staining contrast closer to the real images.

Regarding cellular arrangement and overall tissue structure, hepatocytes in real H&E images usually show a relatively regular arrangement pattern. Some methods may introduce local structural discontinuity or slight arrangement shifts during generation. In comparison, SCGP-CGAN can better maintain the continuity of cellular arrangement and the overall structural relationship in most regions, resulting in higher spatial consistency of the generated results.

Overall, different methods exhibit varying degrees of trade-off between tone mapping and structural preservation. Compared with other methods, SCGP-CGAN shows relatively stable performance in balancing staining expression and structural consistency, indicating its good adaptability for the MT-to-H&E virtual staining task.

### 3.3. Ablation Study

#### 3.3.1. Ablation Study of Skip-Connection Fusion Strategies.

To evaluate the impact of different skip fusion strategies on structural fidelity and reconstruction quality, three commonly used feature fusion strategies were incorporated into the baseline CycleGAN generator for comparative analysis: Concat (feature concatenation) [19], Add-raw (element-wise addition), and Add-conv-align (convolution-aligned addition). Among these, Concat fuses encoder and decoder features along the channel dimension, which helps preserve information and enhance multi-scale representation. In contrast, the addition-based strategies perform cross-layer fusion through element-wise operations, maintaining semantic consistency while avoiding excessive channel redundancy. Experimental results indicate that Concat achieves better performance in terms of information preservation and multi-scale representation, and is therefore adopted as the default configuration. The effects of the three strategies on structural detail preservation and generation stability are further analyzed through ablation experiments. Table 2 summarizes the experimental settings, and Table 3 reports the SSIM and PSNR results for the generated and reconstructed images [41].

**Table 2:** Ablation study on skip-connection feature fusion strategies.

Fusion Type	Implementation Description	Experimental Purpose
No-Skip (Baseline)	No skip connections	To evaluate the improvement contributed by skip connections.
Concat	Feature concatenation	To preserve richer semantic and spatial information.
Add-Raw	Direct element-wise addition	To achieve simple fusion and enhance structural detail preservation.
Add-Concalign	Convolution-aligned addition	To improve structural consistency and edge sharpness.

**Table 3:** Comparison of SSIM and PSNR across different skip-connection feature fusion strategies for generated and reconstructed images.

Fusion Type	Generated Image	Reconstructed Image
No-Skip (Baseline)	SSIM = 0.924、PSNR = 15.21	SSIM = 0.992、**PSNR = 34.94**
Concat	**SSIM = 0.936、PSNR = 16.39**	**SSIM = 0.993、**PSNR = 34.70
Add-Raw	SSIM = 0.905、PSNR = 15.62	SSIM = 0.987、PSNR = 32.35
Add-Conv-Align	SSIM = 0.927、PSNR = 15.73	SSIM = 0.986、PSNR = 32.21

As shown in Table 3, compared with the original generator without skip connections, all three fusion strategies improve both SSIM and PSNR. Among them, the Concat strategy achieves the highest SSIM (0.936) and PSNR (16.39) for the generated images, indicating that feature concatenation helps preserve structural information while maintaining a certain level of detail. It is worth noting that the PSNR values of the generated images are relatively low, which is commonly observed in unsupervised image-to-image translation tasks. This is mainly because PSNR measures pixel-level consistency, whereas generated images may exhibit perceptible differences in color, texture, or local structure. Previous studies have shown that pixel-level metrics such as PSNR have limited correlation with human visual perception [23]. In addition, GAN-based methods tend to prioritize visual realism over strict pixel-wise accuracy [16], which further contributes to lower PSNR values in such tasks. To provide a more comprehensive evaluation, SSIM is also employed to assess the structural fidelity and visual quality of the generated images [21].

In terms of image reconstruction, the original generator achieves higher SSIM (0.993) and PSNR (34.94), indicating that cycle consistency contributes to maintaining global structural integrity. The Add-raw and Add-conv-align strategies yield SSIM values of 0.987 and 0.986, respectively, with corresponding PSNR values of 32.35 and 32.21. Although slightly lower, these results remain at a relatively high level overall. Considering both generation and reconstruction performance, the Concat strategy provides a more balanced trade-off between detail preservation and structural restoration, and is therefore selected as the default fusion approach.

#### 3.3.2. Ablation study of skip-connection configurations.

To further analyze the role of skip connections and their interaction with the residual modules, ablation studies were conducted on different skip connection configurations after adopting Concat as the default fusion method. Three structures were considered: no skip connections (Baseline), partial skip connections, and full skip connections. In the Partial Skip setting, feature fusion is applied only between shallow and intermediate layers, whereas in the Full Skip configuration, feature concatenation is introduced across all corresponding encoder–decoder layers.

Table 4 presents the quantitative results for different skip connection configurations. As indicated in the table, these experiments are conducted without incorporating the CBAM attention mechanism or the loss combination, allowing the effect of skip connections to be evaluated independently. Compared with the baseline model without skip connections, all metrics show improvements after introducing skip connections. Partial skip connections increase SSIM (0.9239–0.9241 to 0.9279–0.9281) and PSNR (15.13–15.29 to 15.80–15.94 dB), while LPIPS decreases from 0.175 to 0.172 and FID from 85.12 to 82.11, indicating improved perceptual quality and distribution consistency. Building on this, full skip connections further enhance performance, raising SSIM to 0.9359–0.9361 and PSNR to approximately 16.46 dB, while reducing LPIPS and FID to 0.171 and 80.96, respectively. MS-SSIM (0.848) and FSIM (0.866) also reach their highest values. These results suggest that multi-level skip connections facilitate structural information transfer and improve detail representation.

**Table 4:** Ablation results for different skip connection configurations.

Model	CBAM attention mechanism	Loss Combination	Skip Configuration	SSIM↑	PSNR↑	LPIPS↓	FID↓	MS-SSIM↑	FSIM↑
Cyclegan	None	None	None	(0.9239,0.9241)	(15.131,15.289)	0.175	85.12	0.827	0.834
Cyclegan+skip	None	None	Partial(e2,e1)	(0.9279,0.9281)	(15.797,15.943)	0.172	82.11	0.831	0.859
Proposed Model	None	None	Partial(e3,e2,e1)	(0.9359,0.9361)	(16.323,16.457)	0.171	80.96	0.848	0.866

Overall, by integrating multi-scale features, skip connections improve structural detail and overall generation quality without weakening deep feature modeling. They also complement the residual modules, enabling a balanced trade-off between structural fidelity and perceptual quality.

#### 3.3.3. Ablation Study and Performance Analysis of Attention Mechanisms.

Introducing skip connections improves structural preservation and detail representation in the generated images; however, issues such as uneven local color distribution and limited texture contrast remain. To further evaluate the effects of attention mechanisms within the virtual staining framework, two widely used lightweight attention modules, SE [44] and CBAM, were incorporated into the generator for comparative analysis. The results indicate that CBAM is more effective for modeling multi-scale tissue features. Accordingly, it is embedded in the 2nd–4th residual blocks of the generator to facilitate contextual integration and enhance local feature representation during the transition between encoding and decoding, while maintaining manageable computational cost. To further assess the effects of attention type and insertion position, ablation experiments are conducted. The corresponding configurations and results are presented in Table 5. These experiments mainly assess the relative contributions of skip connections and attention mechanisms within the integrated framework under a unified training setup, rather than introducing new architectural components. The results mainly reflect the relative impacts of different structural designs on model performance. The results of the final model with the complete loss function design will be uniformly presented in the subsequent subsections.

**Table 5:** Ablation study of attention mechanisms.

Model Configuration (Mechanism Type and Insertion Position)	SSIM↑	PSNR↑	LPIPS↓	FID↓	MS-SSIM↑	FSSIM↑
None (Baseline)	(0.9359,0.9361)	(16.323,16.457)	0.171	60.96	0.848	0.866
SE (Res-All)	(0.9499,0.9501)	(17.169,17.251)	0.195	59.14	0.851	0.855
SE (Res-234)	(0.9289,0.9291)	(18.077,18.223)	0.180	60.52	0.870	0.863
SE (Res-234 + Last)	(0.9419,0.9421)	(17.129,17.231)	0.182	57.41	0.874	0.878
SE (Res-234 + Skip-All)	(0.9269,0.9271)	(17.964,18.076)	0.154	59.09	0.882	0.896
CBAM (Res-All)	(0.9529,0.9531)	(18.844,18.916)	0.156	50.79	0.912	0.901
CBAM (Res-234)	**(0.9759,0.9761)**	**(23.696,23.904)**	**0.167**	**49.08**	**0.934**	**0.928**
CBAM (Res-234 + Last)	(0.9429,0.9431)	(18.402,18.598)	0.162	50.28	0.905	0.897
CBAM (Res-234 + Skip-All)	(0.9479,0.9481)	(17.670,17.770)	0.157	54.61	0.929	0.917

As shown in Table 5, the introduction of the attention mechanism led to differentiated changes across model configurations under different evaluation metrics. Compared with the baseline model, most configurations show improvements in SSIM, PSNR, MS-SSIM, and FSIM, whereas LPIPS and FID exhibit different trends. This reflects a trade-off among structural consistency, perceptual similarity, and distribution alignment. This behavior can be attributed to the differing optimization objectives of these metrics. Specifically, SSIM and PSNR emphasize structural and pixel-level consistency, while LPIPS focuses more on high-level semantic features and perceptual differences. When the SE module is applied to all residual blocks (Res-All), SSIM, PSNR, and MS-SSIM increase relative to the baseline. However, LPIPS rises to 0.195 and FSIM shows a slight decrease, indicating that improvements in structural consistency are not accompanied by corresponding gains in perceptual similarity. When SE is applied only to mid-to-high layers (Res-234 or Res-234 + Last), FID decreases, but MS-SSIM and FSIM exhibit fluctuations, and LPIPS does not reach its lowest value. This suggests that channel attention alone has limitations in modeling complex textures. Furthermore, although SE (Res-234 + Skip-All) achieves the lowest LPIPS (0.145), its SSIM, PSNR, and MS-SSIM are not optimal, highlighting the discrepancy between structural and perceptual evaluation metrics.

In comparison, CBAM demonstrates more stable performance across structural and distribution-related metrics. When embedded in the mid-level residual blocks (Res-234), the model achieves the highest SSIM (0.9759–0.9761) and PSNR (23.696–23.904), along with the lowest FID (49.08). It also attains relatively high values for MS-SSIM (0.934) and FSIM (0.928), indicating strong performance in structural preservation, multi-scale consistency, and distribution alignment. However, its LPIPS value (0.167) is not the lowest, suggesting that improvements in structure-related metrics do not fully align with perceptual similarity measures. Further extending CBAM to all layers or combining it with skip connections does not lead to additional gains, indicating that the placement of attention modules requires a balance between feature representation and redundancy. Therefore, Res-234 was selected as the final generator configuration based on its overall performance across multiple complementary evaluation metrics rather than on a single metric.

#### 3.3.4. Performance Analysis of the Model Under Multi-Loss Constraints.

Building upon the introduction of skip connections and the CBAM attention module, this study further incorporates multiple loss constraints during training. These constraints are designed to optimize structural consistency, perceptual similarity, and distribution alignment in the generated results. The generator includes SSIM, LPIPS, style, and color losses, while the discriminator employs the WGAN-GP adversarial loss to optimize distribution alignment between real and generated images. To analyze the contribution of each loss term, ablation experiments are conducted by sequentially removing individual components (see Table 6).

**Table 6:** Ablation study on loss components and training strategy (SCGP-CGAN).

Model	SSIM↑	PSNR↑	LPIPS↓	FID↓	MS-SSIM↑	FSIM↑
CycleGAN+Skip Connection+CBAM Attention(Baseline)	(0.9759,0.9761)	(23.696,23.904)	0.167	49.08	0.934	0.928
Baseline+WGAN-GP+SSIM+LPIPS+Style	(0.9459,0.9461)	(17.621,17.799)	**0.116**	35.20	0.928	0.935
Baseline+Color+SSIM+LPIPS+Style	(0.9039,0.9041)	(11.778,12.122)	0.156	50.85	0.874	0.898
Baseline+Color+WGAN-GP +LPIPS+Style	(0.9109,0.9111)	(15.244,15.516)	0.149	49.33	0.911	0.902
Baseline+Color+WGAN-GP+SSIM+Style	(0.9419,0.9421)	(17.134,17.366)	0.144	33.68	0.944	0.926
Baseline+Color+WGAN-GP+SSIM+LPIPS	(0.9799,0.9801)	(22.123,22.817)	0.146	31.80	0.944	0.932
Baseline+Color+WGAN-GP+SSIM+LPIPS+Style(SCGP-CGAN)	**(0.9829,0.9831)**	**(24.277,24.703)**	0.138	**27.41**	**0.956**	**0.941**

As shown in Table 6, the complete model demonstrates relatively stable performance across multiple metrics (SSIM: 0.9829–0.9831, PSNR: 24.277–24.703, FID: 27.41, MS-SSIM: 0.956, FSIM: 0.941). The full model achieves a balanced overall performance across the evaluated metrics, indicating that the different loss components provide complementary constraints on structural consistency, perceptual similarity, and distribution alignment. In some ablation settings, performance drops below the baseline due to conflicting optimization objectives among loss terms, which may lead to suboptimal convergence when certain constraints are removed. These results indicate that joint optimization of multiple loss terms helps maintain structural fidelity, perceptual similarity, and distribution consistency. When WGAN-GP is removed, SSIM and PSNR decrease to 0.9039–0.9041 and 11.778–12.122, respectively, while FID increases to 50.85. At the same time, MS-SSIM (0.874) and FSIM (0.898) show noticeable declines, suggesting that adversarial distribution constraints contribute to distribution alignment and the overall generation quality. Similarly, removing the color loss leads to an increase in FID (35.20) and a decrease in PSNR (17.621–17.799), with MS-SSIM (0.928) and FSIM (0.935) also lower than those of the complete model. This indicates that color constraints contribute not only to staining consistency but also to maintaining structural and feature similarity.

There is a trade-off between evaluation metrics associated with structural and perceptual losses. After removing the SSIM loss, structural similarity decreases to 0.9109–0.9111, and PSNR drops to 15.244–15.516. MS-SSIM (0.911) and FSIM (0.902) also decline, indicating that SSIM contributes to preserving structural information across multiple scales. In contrast, removing LPIPS results in relatively small changes in structural metrics (SSIM = 0.9419–0.9421, MS-SSIM = 0.944, FSIM = 0.926), while FID increases to 33.68. This suggests that perceptual constraints primarily influence the distribution of high-level features. Overall, LPIPS emphasizes high-level perceptual feature alignment [23], whereas SSIM, MS-SSIM, and PSNR focus on structural consistency [21]. A trade-off therefore exists between perceptual quality and distortion [45]. As a result, the model may prioritize structural constraints during optimization, which can affect high-level semantic consistency and distribution alignment. This also explains the observed increase in FID after removing LPIPS.

For the ablation of the style loss, only moderate changes are observed across the evaluation metrics, with SSIM within 0.9799–0.9801 and PSNR within 22.817–22.123 dB, while FID is 31.80. Meanwhile, MS-SSIM (0.944) and FSIM (0.932) are also slightly lower than those of the full model. These results indicate that the style constraint mainly plays a complementary role. It helps regulate texture representation and staining hierarchy. However, its overall influence is smaller than that of the adversarial, structural, or perceptual losses.

Overall, the joint optimization of multiple loss functions does not uniformly improve all evaluation metrics. Instead, it achieves a balance between structural consistency, perceptual similarity, and distribution alignment. Based on this performance, subsequent experiments employed a configuration incorporating all loss terms as the default training scheme.

#### 3.3.5. Cumulative Contribution of Each Component.

The ablation results show that the model performance improves as different components are progressively introduced.

As shown in Table 7, taking CycleGAN as the baseline, the inclusion of skip connections leads to improvements across all metrics, indicating their role in feature propagation and information preservation. With the further introduction of the CBAM attention mechanism, the model performance improves again. Specifically, SSIM increases from approximately 0.936 to 0.976, and PSNR rises from around 16.4 dB to over 23.8 dB. Meanwhile, FID decreases from 80.96 to 49.08, and LPIPS is also reduced. These results suggest that the attention mechanism contributes to modeling structural and texture information. Finally, after incorporating the combined loss functions, the model performance continues to improve. SSIM and MS-SSIM reach 0.983 and 0.956, respectively, while PSNR further increases and FID decreases to 27.41. LPIPS also shows a reduction, indicating an improvement in perceptual quality.

**Table 7:** Quantitative comparison of ablation experiments under different model configurations. The results show the progressive improvement of image quality as key components are incrementally introduced, including skip connections, CBAM attention, and combined loss functions. Metrics include SSIM, PSNR, LPIPS, FID, MS-SSIM, and FSIM.

Model	SSIM↑	PSNR↑	LPIPS↓	FID↓	MS-SSIM↑	FSIM↑
Baseline(CycleGAN)	(0.9239,0.9241)	(15.131,15.289)	0.175	85.12	0.827	0.834
Baseline+Skip Connection	(0.9359,0.9361)	(16.323,16.457)	0.171	80.96	0.848	0.866
Baseline+Skip Connection+CBAM Attention	(0.9759,0.9761)	(23.696,23.904)	0.167	49.08	0.934	0.928
Baseline+Skip Connection+CBAM Attention+ Loss Combination(SCGP-CGAN)	**(0.9829,0.9831)**	**(24.277,24.703)**	**0.138**	**27.41**	**0.956**	**0.941**

Overall, each component has an impact on model performance, and the complete model shows a more balanced performance in terms of structural preservation, perceptual quality, and distribution consistency.

#### 3.3.6. Sensitivity Analysis of Loss Weights.

The loss weights were selected with reference to related studies and empirical evaluation of different weight settings. The selection considered the contributions of the individual loss terms to structural preservation, perceptual similarity, color consistency, and style representation. Based on these experiments, the final weights were set to $\lambda_{\text{SSIM}}$ = 0.15, $\lambda_{\text{LPIPS}}$ = 0.2, $\lambda_{\text{color }}$ = 3.0, and $\lambda_{\text{style}}$ = 1.5. Sensitivity analyses were further conducted for $\lambda_{\text{SSIM}}$ and $\lambda_{\text{LPIPS}}$ with the other loss weights fixed, to examine their effects on the overall performance.

Table 8 shows that, with other loss weights held constant ($\lambda_{\text{LPIPS}}$ = 0.2, $\lambda_{\text{color }}$ = 3.0, $\lambda_{\text{style}}$ = 1.5), varying $\lambda_{\text{SSIM}}$ impacts model performance. When $\lambda_{\text{SSIM}}$ = 0.15, the model achieves the best overall performance. At this setting, SSIM falls within the range of 0.9829–0.9831 and PSNR ranges from 24.277 to 24.703 dB. Meanwhile, LPIPS (0.138) and FID (27.41) attain their lowest values. In addition, MS-SSIM (0.956) and FSIM (0.941) also reach their highest levels, suggesting that this configuration provides improved stability in multi-scale structural consistency and feature similarity. These results indicate that the generated images maintain strong structural consistency and overall quality. When $\lambda_{\text{SSIM}}$ is set to 0.10 or 0.20, all metrics decline, indicating that overly small or large structural constraints can disrupt the model’s balance between structural fidelity and perceptual quality.

**Table 8:** Sensitivity analysis of SSIM loss weights, with the other loss weights held constant at $\lambda_{LPIPS}$ = 0.2, $\lambda_{color\;}$ = 3.0, and $\lambda_{style}$ = 1.5.

$\lambda_{𝐒𝐒𝐈𝐌}$	SSIM↑	PSNR↑	LPIPS↓	FID↓	MS-SSIM↑	FSIM↑
0.10	(0.9789,0.9791)	(22.995,23.265)	0.147	29.13	0.947	0.936
0.15	**(0.9829,0.9831)**	**(24.277,24.703)**	**0.138**	**27.41**	**0.956**	**0.941**
0.20	(0.9679,0.9681)	(21.730,21.910)	0.151	30.61	0.938	0.932

Table 9 presents the sensitivity analysis results for $\lambda_{\text{LPIPS}}$. With other loss weights held constant, the model achieves optimal performance when $\lambda_{\text{LPIPS}}$ = 0.2. At this setting, SSIM falls within the range of 0.9829–0.9831 and PSNR ranges from 24.277 to 24.703 dB. Meanwhile, LPIPS (0.138) and FID (27.41) attain their lowest values. MS-SSIM (0.956) and FSIM (0.941) also reach their peak values, indicating that this configuration provides a balanced performance between structural fidelity and perceptual quality. When the weight is decreased to 0.10 or increased to 0.30, all metrics decline to varying degrees, suggesting that both underweighting and overweighting perceptual similarity can adversely affect the balance between structural preservation and perceptual realism.

**Table 9:** Sensitivity analysis of LPIPS loss weights was conducted, with the other loss weights held at their default values.

$\lambda_{𝐋𝐏𝐈𝐏𝐒}$	SSIM↑	PSNR↑	LPIPS↓	FID↓	MS-SSIM↑	FSIM↑
0.10	(0.9469,0.9471)	(19.287,19.453)	0.140	31.20	0.940	0.936
0.20	**(0.9829,0.9831)**	**(24.277,24.703)**	**0.138**	**27.41**	**0.956**	**0.941**
0.30	(0.9539,0.9541)	(17.818,18.142)	0.154	32.45	0.945	0.938

Experiments indicate that the model performs optimally at $\lambda_{\text{SSIM}}$ = 0.15 and $\lambda_{\text{LPIPS}}$ = 0.2, and exhibits relative stability within this range.

### 3.4. Model complexity and inference performance analysis

#### 3.4.1. Model size and single-generator inference analysis.

Table 10 compares CycleGAN and SCGP-CGAN in terms of model size and single-generator inference overhead, measured based on the forward pass of a single generator. After introducing skip connections and CBAM, the number of parameters in SCGP-CGAN increases from 9.50 M to 11.83 M (≈24.4%), while the model size grows from 38.27 MB to 45.14 MB (≈18%). In terms of inference efficiency, the inference time for both G_A and G_B increases from approximately 16.2 ms to 19.1 ms (≈18%). Regarding memory usage, G_A increases from 0.13 GB to 0.19 GB (≈46%), while G_B rises from 0.15 GB to 0.34 GB (≈127%). Overall, although the structural modifications introduce additional computational overhead—particularly the increased memory usage of G_B—the overall increase remains within an acceptable range, and the model maintains a reasonable level of inference efficiency.

**Table 10:** Model parameters and single-generator inference overhead: CycleGAN vs. SCGP-CGAN.

Model	Generator	Params (M) ↓	Model Size (MB) ↓	Inference Time (ms) ↓	GPU Memory (GB)↓
CycleGAN	G_A	9.50	38.27	16.20	0.13
CycleGAN	G_B	9.50	38.27	16.25	0.15
SCGP-CGAN	G_A	11.83	45.14	19.13	0.19
SCGP-CGAN	G_B	11.83	45.14	19.14	0.34

#### 3.4.2. Overall model inference and hardware resource analysis.

Table 11 reports benchmark results for the complete model under the same hardware conditions, including GPU/CPU latency, hardware utilization, and peak VRAM usage during inference. Compared with CycleGAN, SCGP-CGAN shows increased GPU latency (85.20 ms to 128.29 ms) and CPU latency (18.76 ms to 22.97 ms). GPU utilization rises from 30.82% to 45.91%, while CPU utilization increases from 3.70% to 4.50%. GPU and CPU utilization are reported as monitoring metrics reflecting hardware workload during inference, where higher values indicate increased resource usage. The maximum VRAM usage also grows from 0.52 GB to 0.84 GB. Overall, SCGP-CGAN requires more computational resources during inference. This trend is consistent with the increased parameter count and memory overhead observed at the single-generator level. As the results in this table reflect the full model, the reported values are higher than those measured for an individual generator.

**Table 11:** Benchmark tests were performed on CycleGAN and SCGP-CGAN under identical hardware conditions to compare inference latency, CPU and GPU utilization, and memory consumption.

Model	GPU Latency (ms) ↓	CPU Latency (ms) ↓	GPU Util	CPU Util	Max VRAM(MB) ↓
CycleGAN	85.20	18.76	30.82%	3.70%	0.52
SCGP-CGAN	128.29	22.97	45.91%	4.50%	0.84

### 3.5. Limitations Analysis

In the H&E-to-MT virtual staining task, the main limitations of the model lie in the restoration of nuclear features and the consistency of overall color tone.

As shown on the left side of Fig 10, the nuclei in real MT staining exhibit clear boundaries and a dark purple color, while the generated results show relatively blurred nuclear boundaries and insufficient staining contrast. Combined with the magnified views in Fig 11, it can be observed that the model is able to roughly recover the morphological contours of the nuclei. However, the staining intensity remains weak, and the distinction from the surrounding cytoplasm is reduced. This affects the identifiability of cell-level structures. In addition, the annotated regions marked “Overall color tone deviation” indicate that the generated MT images of some samples have an overall purplish hue, with insufficient expression of the typical blue signal of collagen fibers, showing a significant difference from the red-blue contrast feature in real staining. This suggests that the model’s control of staining intensity in complex tissue regions is still not sufficiently stable.

**Fig 10:**
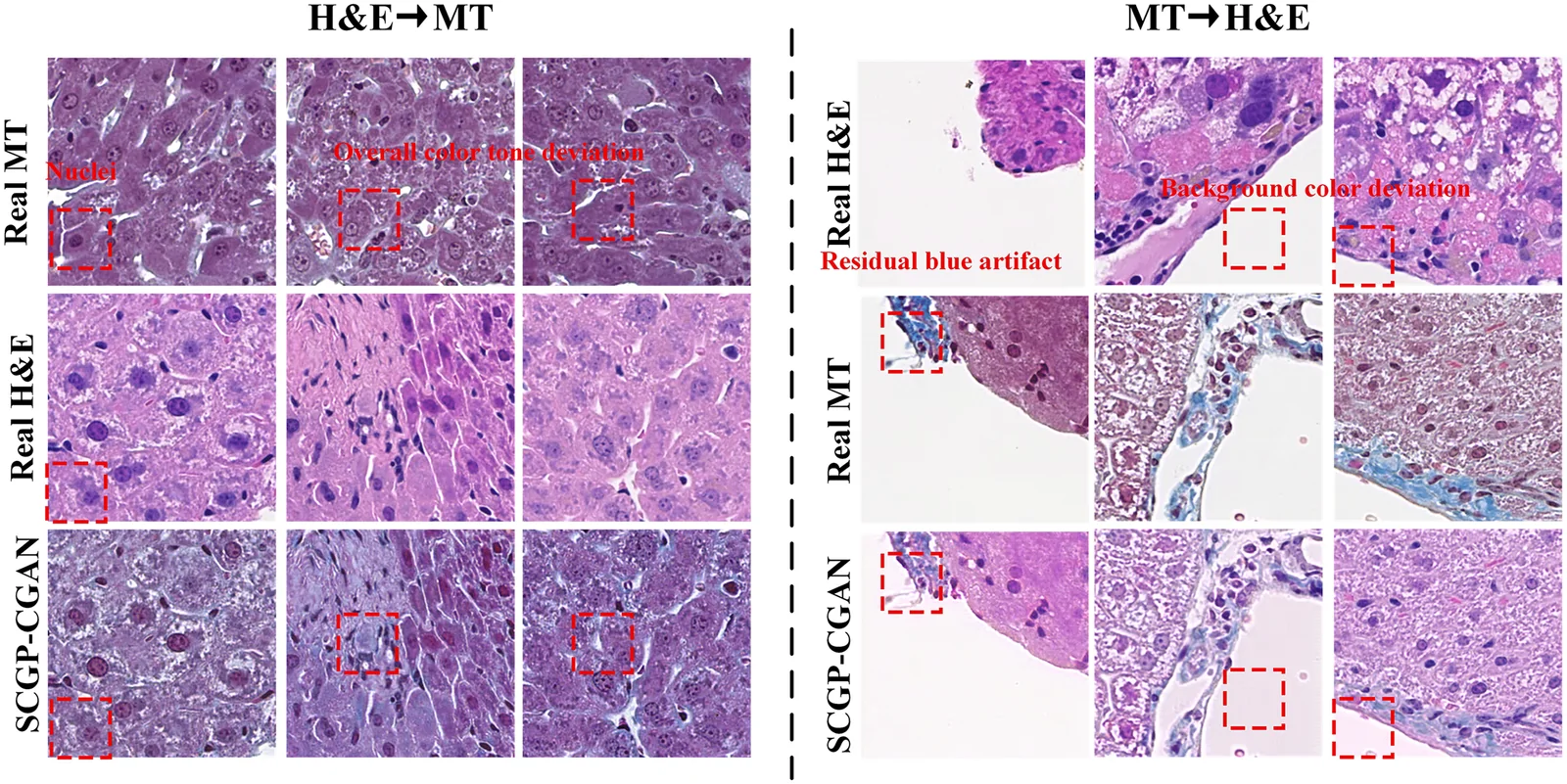
Examples of suboptimal SCGP-CGAN results in bidirectional virtual staining, illustrating the model’s current limitations.

**Fig 11:**
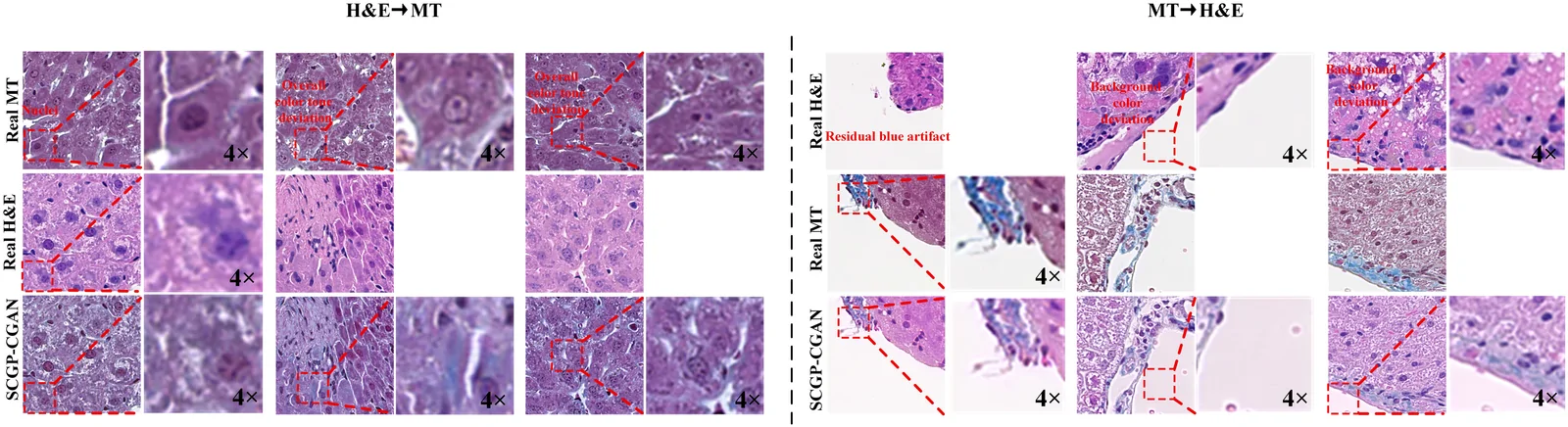
4×  magnified views of the annotated regioiews of the annotated regions in Figure 10, highlighting suboptimal SCGP-CGAN results.

In the MT-to-H&E virtual staining task, the limitations of the model are mainly manifested as residual edge artifacts and background color distortion. As shown on the right side of Fig 10, the blue collagen signals at the tissue edges in real MT staining should appear as light pink to light purple after conversion to H&E staining. However, the generated results still contain regions annotated as “Residual blue artifact”, indicating that some blue information has not been sufficiently removed. Combined with the magnified views in Fig 11, such artifacts are more obvious in the tissue edge regions, affecting the overall visual naturalness. Furthermore, the regions marked “Background color deviation” show that at the junction of tissue and background, the generated H&E images exhibit uneven color tone and local purplish bias, which are inconsistent with the uniform and low-interference background features in real H&E staining. This indicates that the model still has certain errors in tissue-background separation and the restoration of non-signal regions.

Taken together, these unsatisfactory generation results are mainly concentrated in tissue boundary regions, regions with sparse collagen fiber distribution, and regions with complex staining signals. These observations indicate that SCGP-CGAN still has certain limitations in edge detail modeling and global staining consistency. Although the model can achieve relatively stable staining conversion performance on most samples, the above issues indicate that its robustness in complex pathological scenarios still needs improvement. In addition, there remains a certain gap between its current performance and the requirements for clinical diagnostic-level applications. Future research can further optimize the model’s staining reconstruction ability in complex regions by introducing edge-aware constraints, multi-scale feature constraints, or multi-center data training.

## 4. Discussion

This study integrates established architectural and optimization techniques into a task-oriented framework for unpaired H&E–MT virtual staining. The framework combines complementary components to preserve tissue structures, maintain perceptual and color consistency, and improve the stability of adversarial training during bidirectional stain translation. Quantitative comparisons and ablation experiments were conducted to evaluate the overall performance of the framework and the effects of its major components.

While SCGP-CGAN outperforms baseline methods on several metrics, it still has certain limitations. The introduction of skip connections and CBAM increases the number of parameters from 9.50 M to 11.83 M, with the model size growing by approximately 18%. The inference time for a single 256 × 256 image increases from approximately 16.2 ms to 19.1 ms, and memory usage rises by about 0.1–0.2 GB. Despite this increased complexity, the inference efficiency remains within an acceptable range. Further reductions in computational cost may be achieved through model pruning, quantization, or lightweight attention mechanisms.

This study was conducted using a public patch-level dataset derived from a single source under fixed experimental settings. However, the dataset lacks sufficient whole-slide image (WSI)-level or specimen-level metadata to support independent WSI-based validation or multi-center analysis. Therefore, the current experiments primarily evaluate the performance of SCGP-CGAN on the available dataset under the specified experimental conditions. In particular, variations in scanners, staining protocols, tissue-preparation procedures, and specimen characteristics may affect virtual staining performance. Further evaluation on independent datasets and clinical cohorts is needed to assess its robustness and generalizability across different data sources and clinical settings.

An important limitation of the current study is the lack of task-specific downstream validation. Although the generated images were qualitatively assessed by pathologists with respect to key histological structures, such visual assessment does not directly establish whether the generated images preserve or improve performance in downstream histopathological tasks. Future studies should incorporate task-specific validation, such as nuclei detection and counting, tissue or cell segmentation, fibrosis assessment, histopathological classification, and diagnostic concordance analysis, using larger and more diverse datasets. Multi-center evaluation would also be valuable for further assessing the generalizability and practical applicability of the proposed virtual-staining framework. The loss weights used in the integrated objective function are manually specified and may not be optimal for all training scenarios. Future studies could investigate dynamic weighting strategies or meta-learning approaches for more adaptive optimization. Overall, the integrated SCGP-CGAN framework demonstrates satisfactory visual quality and structural representation for bidirectional H&E–MT virtual staining. However, there remains room for improvement in model lightweighting, cross-domain generalization, and adaptive loss optimization. Future work may further validate the model on larger and more diverse datasets, and assess its practical applicability in combination with downstream tasks.

## 5. Conclusion

This study investigates unpaired virtual staining based on the CycleGAN framework by integrating several established structural optimization and loss-design strategies for H&E and MT translation tasks. Skip connections are introduced into the generator to enable multi-scale feature fusion, and a CBAM attention module is embedded within the intermediate residual blocks to enhance the representation of key tissue regions. In terms of loss design, SSIM, style, and color constraints are jointly applied to reinforce structural and stylistic consistency. Meanwhile, a WGAN-GP–based critic is adopted in the discriminator as the adversarial learning strategy.

Experimental results indicate that the integrated framework improves structural preservation and color consistency in bidirectional H&E–MT translation compared with the baseline methods under the evaluated experimental conditions. Further validation on independent and more diverse datasets is needed to assess the robustness and generalizability of the framework. The present study primarily focuses on methodological development and image-level virtual staining performance under the current experimental setting. Frequency-domain information may provide complementary cues for preserving fine structural details in histological images, and frequency-aware methods such as wavelet decomposition or frequency-domain attention could be explored in future work. Further investigation using larger-scale clinical datasets and multi-center validation will be necessary to assess its potential diagnostic applicability and clinical utility.

Overall, the experimental results support the effectiveness of the task-oriented integration of established architectural and optimization components for bidirectional H&E–MT virtual staining. The ablation experiments further indicate that the major components contribute to different aspects of structural and perceptual quality, while the current framework remains subject to limitations in computational efficiency, cross-domain generalization, and adaptive loss optimization. Future work may further validate the framework on larger and more diverse datasets and assess its practical applicability in conjunction with downstream histological analysis tasks.

## Supporting information

S1 Table Direction-specific quantitative results for H&E → MT and MT → H&E translation across different methods. (DOCX)
