# Supplementary material for: Development of a SCGP-CGAN framework for bidirectional H&E–Masson’s Trichrome Virtual Staining
Source: PLoS One. 2026 Nov 5;21(11):e0361713. doi: 10.1371/journal.pone.0361713 (PMC13739232; doi:10.1371/journal.pone.0361713)
Supplement: Supplementary file 1 [file pone.0361713.s001.docx]

As shown in S1 Table, SCGP-CGAN achieves consistently higher SSIM, PSNR, MS-SSIM, and FSIM values and lower LPIPS and FID values than the conventional stain normalization methods and GAN-based image-to-image translation baselines in both H&E→MT and MT→H&E directions. The results indicate that SCGP-CGAN maintains strong structural and perceptual consistency across both translation directions. Although the quantitative performance differs slightly between the two directions, the overall results demonstrate that the proposed framework performs effectively for bidirectional H&E–MT translation.

**S1 Table.** Direction-specific quantitative results for H&E → MT and MT → H&E translation across different methods.

| Method category | Model | SSIM↑ | PSNR↑ | LPIPS↓ | FID↓ | MS-SSIM↑ | FSIM↑ |
| --- | --- | --- | --- | --- | --- | --- | --- |
| Conventional stain normalization | Macenko | (0.8900,0.8901)  (0.9020,0.9021) | (14.543,14.917)  (14.781,15.119) | 0.185  0.197 | 96.14 | 0.819 | 0.818 |
|  | Vahadane | (0.8902,0.8904)  (0.8876,0.8878) | (13.878,14.198)  (14.050,14.354) | 0.195  0.203 | 98.56  97.94 | 0.812  0.796 | 0.808  0.812 |
| GAN-based image-to-image translation | CycleGAN | (0.9231,0.9234)  (0.9247,0.9248) | (15.102,15.110)  (15.160,15.468) | 0.171  0.179 | 82.99  87.25 | 0.831  0.823 | 0.840  0.828 |
|  | StainGAN | (0.9201,0.9203)  (0.9197,0.9199) | (14.765,15.121)  (14.797,15.117) | 0.178  0.190 | 87.96  88.30 | 0.819  0.831 | 0.822  0.814 |
|  | CUT | (0.9225,0.9226)  (0.9213,0.9216) | (14.796,15.121)  (15.000,15.363) | 0.183  0.175 | 87.41  86.17 | 0.834  0.816 | 0.821  0.837 |
|  | SCGP-CGAN | **(0.9825,0.9828)**  **(0.9833,0.9834)** | **(24.130,24.579)**  **(24.424,24.827)** | **0.135**  **0.141** | **28.35**  **26.47** | **0.947**  **0.965** | **0.938**  **0944** |
